# Effects of ribonucleotides on telomeric G4 formation, dynamics, and initiation of ribonucleotide excision repair by RNase H2

**DOI:** 10.1093/nar/gkaf1501

**Published:** 2026-01-14

**Authors:** Luis M Cortez, Md Ibnul Rifat Rahman, Griffin A Welfer, Fillipo Riva, Kristen J Buettner, Hui-Ting Lee, Bret D Freudenthal

**Affiliations:** Department of Biochemistry and Molecular Biology and Department of Cancer Biology, University of Kansas Medical Center, Kansas City, KS 66160, United States; Department of Chemistry, University of Alabama at Birmingham, Birmingham, AL 35294, United States; Department of Biochemistry and Molecular Biology and Department of Cancer Biology, University of Kansas Medical Center, Kansas City, KS 66160, United States; Department of Biochemistry and Molecular Biology and Department of Cancer Biology, University of Kansas Medical Center, Kansas City, KS 66160, United States; Department of Chemistry, University of Alabama at Birmingham, Birmingham, AL 35294, United States; Department of Chemistry, University of Alabama at Birmingham, Birmingham, AL 35294, United States; Department of Biochemistry and Molecular Biology and Department of Cancer Biology, University of Kansas Medical Center, Kansas City, KS 66160, United States; University of Kansas Cancer Center, Kansas City, KS 66160, United States

## Abstract

Human telomeres are composed of TTAGGG repeats that can fold into G-quadruplexes (G4s). G4s can form several different conformations, including parallel, antiparallel 2 + 2 chair, antiparallel 2 + 2 basket, and 3 + 1 parallel/antiparallel. Telomeres are composed of deoxyribonucleotide monophosphates; however, telomerase has been shown to insert ribonucleotide monophosphates (rNMPs) as efficiently as replicative DNA polymerases. Non-telomeric rNMP insertions are deleterious, but the effect on telomeres remains under explored. We systematically investigated 16 variants of the G4-forming telomeric sequence (TTAGGG)_4_ containing a single rNMP substitution. We generally found that rNMP substitution of the first dG in a repeat (TTAGGG)_4_ altered the G4 conformation. Incorporation of a rNMP also perturbed G4 folding dynamics, decreasing the population of stably folded molecules and promoting rapid structural transitions. Depending on the rNMP position, we further observed a reduction in overall thermal stability. Additionally, RNase H2, the initiator of ribonucleotide excision repair, had reduced cleavage of rNMPs in G4s, and could only cleave rNMPs at more accessible positions within the G4. Cumulatively, we show that the insertion of rNMPs in telomeric sequences alters the conformation and stability of G4s. This could lead to deleterious effects on telomeric integrity, and these changes may persist due to the difficulty of repairing rNMPs within G4s.

## Graphical Abstract

**Graphical Abstract:**
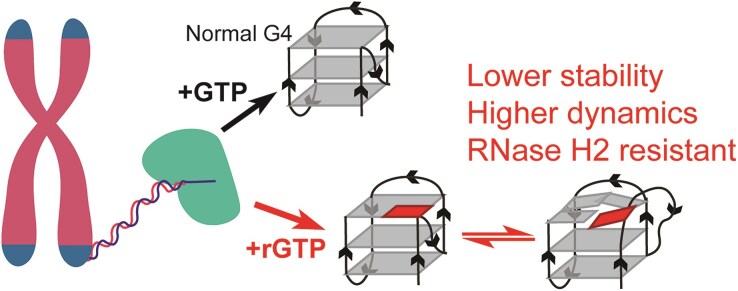

## Introduction

**Figure 1.**
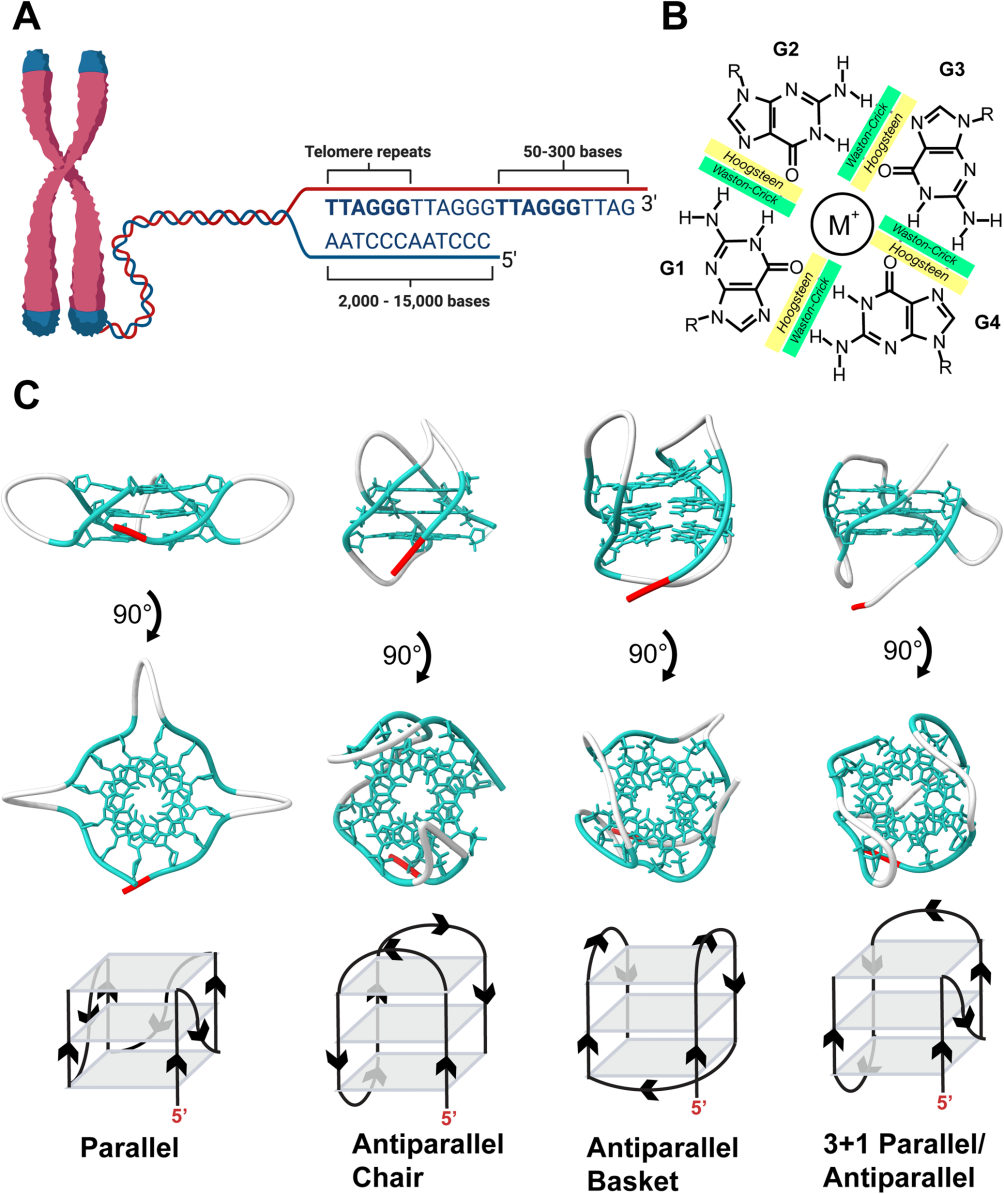
G-quadruplex telomeric structures. (**A**) Depiction of chromosome with bulk genome in red, and telomere ends in blue. The human telomeric sequence is shown with both double-stranded and single-stranded portions indicated. Created in BioRender. Freudenthal, B. (2025) https://BioRender.com/vubef2h. (**B**) G-tetrad schematic with the monovalent cation, Hoogsteen, and Watson–Crick faces indicated. (**C**) Side and top view of various intramolecular G-quadruplex conformations with guanine bases in cyan, the loop regions in white, and 5′ end in red. Also shown are cartoon representations of the G-quadruplexes. PDB ID: 1KF1, 5YEY, 143D, and 2HY9.

Telomeres are nucleoprotein complexes found at the ends of chromosomes that function as protective caps to maintain genomic integrity [1, 2]. Human telomeres are composed of 2–15 kb of tandem noncoding DNA repeats (TTAGGG) that end with a 50–300 base overhang of 3′ single-stranded DNA (ssDNA) (Fig. 1A) [2–7]. Because this overhang can be misrecognized as DNA damage, it must be protected from DNA repair pathways. This “end protection problem” is solved by sequestering the single-stranded telomeric overhang into a displacement loop within the duplex portion of the telomere: this process is modulated by the shelterin complex, a group of telomere associated proteins [8–11]. However, even with this end protection mechanism, some DNA is lost through successive rounds of replication; due to the mechanism of lagging strand DNA synthesis, cells are unable to replicate the terminal end of their DNA [12–14]. Eventually, telomeres shorten to a critical point, after which they can no longer sufficiently bind the shelterin complex, and the cell becomes senescent [15, 16]. However, germline cells, stem cells, and up to 90% of all cancer cells are able to maintain their replicative capacity by preventing the shortening of their telomeres via the reverse transcriptase telomerase [1]. Telomerase binds to the ssDNA 3′ overhang and catalyzes the addition of the TTAGGG telomeric repeats in a processive manner[17, 18].

One strand of the telomeric DNA is rich in the nucleotide guanine (G) and can thus fold into secondary structures known as G-quadruplexes (G4s), which may inhibit telomerase’s ability to extend telomeres [19–22]. G4s form when multiple G tetrads stack on each other [21, 22]. G tetrads are composed of four guanines that hydrogen bond to each other using both their Hoogsteen and Watson–Crick faces (Fig. 1B) [21–23]. The G tetrads are further stabilized by an internal monovalent cation, most commonly potassium, which both coordinates the carbonyl oxygens of the guanine nucleobases and counteracts the negative charge of the phosphate backbone: this allows the DNA to fold into a complete G4 [21, 24–26]. Intramolecular G4s can fold into several different conformations including parallel, antiparallel 2 + 2 chair, antiparallel 2 + 2 basket, and 3 + 1 parallel/antiparallel (Fig. 1C) [27–31].

The presence and conformation of G4s have critical consequences on telomere maintenance, but the conditions under which specific conformations form are poorly understood [32, 33]. Studies using G4 antibodies *in vivo* indicate that telomeric G4s are predominantly folded into parallel structures, but biochemical studies *in vitro* indicate that telomeric G4s are predominately folded into 3 + 1 parallel/antiparallel structures [34–36]. There is further evidence that the parallel structures observed *in vivo* may arise from kinetic partitioning and conformation non-specificity of the G4 antibodies [25, 36–38]. There are several potential explanations for the discrepancies between these studies. Molecular crowding is an aspect of the cellular milieu that is known to affect G4 conformation but that is not typically recapitulated *in vitro* [39–41]. Consistent with this, oligos that form antiparallel G4 in dilute solutions have been shown to instead form parallel G4 under molecular crowding conditions [40–42]. Additionally, *in vivo*, DNA folds as it is synthesized and released by telomerase, rather than all at once as *in vitro*; this may also lead to different conformations [43]. It has thus become clear that there are different factors, such as molecular crowding, DNA sequence, DNA base damage, and metal identity, that contribute to the conformation and the stability of a G4. One such factor that can alter the conformational dynamics of the G4 is the presence of ribonucleotide monophosphates (rNMPs) in place of the canonical deoxynucleotide monophosphates (dNMPs) [32, 33, 44–47].

While the genome is canonically composed of dNMPs, misincorporation of rNMPs may occur during DNA replication. Pol δ will insert one rNMP for every 2000 insertion events; thus, this form of DNA damage occurs on the order of one million rNMPs inserted per cell cycle [48–50]. Incorporation of genomic rNMPs can promote genome instability, alter the DNA helical structure, and disrupt chromatin packaging, which can lead to multiple human diseases [51–57]. Given the deleterious effects of rNMP incorporation into genomic DNA, cells have evolved a repair mechanism called the ribonucleotide excision repair (RER) pathway to remove embedded rNMPs. RER is initiated by the enzyme RNase H2, which recognizes an rNMP embedded in DNA [51, 58]. After recognition of the lesion, RNase H2 nicks the DNA backbone 5′ of the rNMP [59–61]. Pol δ then uses the resulting 3′-OH to perform strand displacement synthesis, which generates a flap containing the excised rNMP [58]. The flap containing the rNMP is then cleaved by the enzyme FEN1, and the nicked DNA product is sealed by DNA ligase I, resulting in error free removal of rNMPs [58].

Telomerase has comparable sugar fidelity as replicative DNA polymerases: as we and others have previously demonstrated, telomerase is able to insert rNMPs, with a rate of insertion up to 1 rNMP for every 280 insertion events under physiological nucleotide concentrations [62, 63]. Using a sequencing-based approach, it was found that human telomeres accumulate higher levels of rNMPs than the rest of the genome, consistent with enhanced incorporation by telomerase and inefficient removal [64]. However, the impact of rNMPs on telomeres and their repair remains poorly explored. To investigate this, we systematically replaced canonical dNMPs with rNMPs in the fundamental G4-forming repeat of human telomeric DNA: (TTAGGG)_4_. We found that when a single rNMP is substituted into a G4-forming repeat, it can induce changes in G4 conformation. A single rNMP substitution also reduces the fraction of molecules that stay folded as a G4 and increases the number of molecules that undergo conformational change in a short time frame. Additionally, insertion of a rNMP at certain critical positions greatly reduces the overall stability of the resulting G4. Finally, we show that RNase H2-mediated cleavage of an embedded rNMP within the G4 structure is critically dependent on the location of the rNMP within the G4. Overall, we report here that rNMP insertion in human telomere repeat sequences reduces the stability of telomeric G4 by altering the conformation and increasing conformational dynamics.

## Materials and methods

### Oligonucleotides

All DNA oligonucleotides (Tables 1 –4) were purchased from Integrated DNA Technologies (IDT). DNA was annealed at 1 mM concentration in annealing buffer (100 mM KCl and 50 mM HEPES, pH = 8.0) at 95°C for 5 min, and gradually cooled for ~4 h to 4°C. Folded G4 samples were then used immediately or stored at −20°C until use.

**Table 1.** G4-forming telomeric sequences used with the location of the ribonucleotide substitution is denoted with lower case r and underlined

Sample	Sequence	Conformation	Melt°C	ΔMelt°C
TEL	TTAGGGTTAGGGTTAGGGTTAGGG	3 + 1	58.86	0
TELR3	TTrAGGGTTAGGGTTAGGGTTAGGG	3 + 1	61.17	2.31
TELR4	TTArGGGTTAGGGTTAGGGTTAGGG	Antiparallel basket, 3 + 1 (290)	55.69	−3.16
TELR4	TTArGGGTTAGGGTTAGGGTTAGGG	Antiparallel basket, 3 + 1 (275)	59.33	0.48
TELR5	TTAGrGGTTAGGGTTAGGGTTAGGG	3 + 1	57.35	−1.51
TELR6	TTAGGrGTTAGGGTTAGGGTTAGGG	3 + 1	59.86	1.01
TELR9	TTAGGGTTrAGGGTTAGGGTTAGGG	3 + 1	60.77	1.92
TELR10	TTAGGGTTArGGGTTAGGGTTAGGG	Antiparallel basket	46.99	−11.87
TELR11	TTAGGGTTAGrGGTTAGGGTTAGGG	3 + 1	57.16	−1.70
TELR12	TTAGGGTTAGGrGTTAGGGTTAGGG	3 + 1	61.42	2.56
TELR15	TTAGGGTTAGGGTTrAGGGTTAGGG	3 + 1	60.34	1.48
TELR16	TTAGGGTTAGGGTTArGGGTTAGGG	Parallel, antiparallel chair (270)	58.33	−0.53
TELR16	TTAGGGTTAGGGTTArGGGTTAGGG	Parallel, antiparallel chair (295)	57.95	−0.91
TELR17	TTAGGGTTAGGGTTAGrGGTTAGGG	3 + 1	57.49	−1.37
TELR18	TTAGGGTTAGGGTTAGGrGTTAGGG	3 + 1	61.38	2.52
TELR21	TTAGGGTTAGGGTTAGGGTTrAGGG	3 + 1	61.26	2.40
TELR22	TTAGGGTTAGGGTTAGGGTTArGGG	3 + 1, parallel	58.37	−0.49
TELR23	TTAGGGTTAGGGTTAGGGTTAGrGG	3 + 1	59.12	0.27
TELR24	TTAGGGTTAGGGTTAGGGTTAGGrG	3 + 1	59.40	0.54

Summary of the results from the CD experiments showing the G4 conformation, melting point, and difference in melting point compared to the DNA only TEL sequence for each G4 substrate is shown.

**Table 2.** Percentage of different types of single-molecule traces for telomeric constructs

Constructs	Folded	Dynamic	Unfolded	Peak Center	Half-Width
TELR9_sm_	73.1 ± 9.43	19.6 ± 2.88	7.28 ± 5.28	0.68 ± 0.01	0.14 ± 0.01
TELR10_sm_	58.8 ± 4.87	34.6 ± 4.87	6.19 ± 3.11	0.68 ± 0.02	0.15 ± 0.01
TELR11_sm_	56.0 ± 3.87	38.9 ± 2.96	5.05 ± 2.68	0.67 ± 0.01	0.14 ± 0.01
TELR12_sm_	65.2 ± 3.05	29.0 ± 4.57	5.74 ± 3.75	0.67 ± 0.01	0.15 ± 0.01
TELR21_sm_	71.8 ± 3.45	25.8 ± 2.7	2.48 ± 1.41	0.66 ± 0.01	0.13 ± 0.01
TELR22_sm_	66.8 ± 2.79	29.2 ± 2.79	3.99 ± 1.84	0.664 ± 0.012	0.14 ± 0.01
TELR23_sm_	54.9 ± 2.48	39.6 ± 1.95	5.51 ± 1.27	0.65 ± 0.01	0.14 ± 0.01
TELR24_sm_	48.2 ± 4.87	46.1 ± 2.36	5.72 ± 4.16	0.68 ± 0.01	0.16 ± 0.01
TEL_sm_	74.3 ± 2.50	17.8 ± 5.13	7.89 ± 3.29	0.67 ± 0.01	0.14 ± 0.01
TEL-TTAG	67.9 ± 1.74	29.6 ± 1.24	2.25 ± 0.69	0.65 ± 0.01	0.12 ± 0.02

**Table 3.** Percentage of RNase H2 cleavage of G4s in the presence of potassium chloride

Sample	Sequence	1 min	5 min	15 min	30 min	60 min	120 min
dsTELR10	TTAGGGTTArGGGTTAGGGTTAGGG	96%	98%	99%	99%	99%	99%
TELR3	TTrAGGGTTAGGGTTAGGGTTAGGG	N.D.	N.D.	N.D.	N.D.	N.D.	N.D.
TELR4	TTArGGGTTAGGGTTAGGGTTAGGG	N.D.	N.D.	N.D.	N.D.	N.D.	N.D.
TELR5	TTAGrGGTTAGGGTTAGGGTTAGGG	N.D.	N.D.	N.D.	N.D.	N.D.	N.D.
TELR6	TTAGGrGTTAGGGTTAGGGTTAGGG	N.D.	N.D.	N.D.	N.D.	N.D.	N.D.
TELR9	TTAGGGTTrAGGGTTAGGGTTAGGG	6.5 ± 1.1%	7 ± 1.8%	6.8 ± 1.1%	6.7 ± 1%	7.6 ± 2.7%	7.4 ± 2.1%
TELR10	TTAGGGTTArGGGTTAGGGTTAGGG	2.4 ± 0.7%	2.9 ± 0.5%	3.8 ± 0.3%	4.5 ± 0.6%	6.3 ± 0.3%	9.8 ± 0.3%
TELR11	TTAGGGTTAGrGGTTAGGGTTAGGG	1.9 ± 1.2%	1.9 ± 1.1%	1.4 ± 0.7%	2.1 ± 1.2%	2.1 ± 1.1%	2.1 ± 1.2%
TELR12	TTAGGGTTAGGrGTTAGGGTTAGGG	0.6 ± 1%	0.8 ± 1.1%	0.7 ± 1%	0.6 ± 1%	1 ± 1.4%	0.7 ± 1.1%
TELR15	TTAGGGTTAGGGTTrAGGGTTAGGG	6 ± 0.1%	6.1 ± 0.2%	6.6 ± 0.3%	6.9 ± 0.3%	8 ± 0.4%	9.4 ± 0.5%
TELR16	TTAGGGTTAGGGTTArGGGTTAGGG	2.3 ± 1.2%	2.5 ± 1.2%	3.3 ± 1.6%	4 ± 1.9%	5.4 ± 2.4%	7.2 ± 2.8%
TELR17	TTAGGGTTAGGGTTAGrGGTTAGGG	1.8 ± 1.1%	1.7 ± 1%	1.9 ± 1%	2.1 ± 1.4%	2.3 ± 1.4%	2.7 ± 1.4%
TELR18	TTAGGGTTAGGGTTAGGrGTTAGGG	0.2 ± 0.3%	N.D.	N.D.	N.D.	0.2 ± 0.3%	0.2 ± 0.4%
TELR21	TTAGGGTTAGGGTTAGGGTTrAGGG	4.6 ± 2.2%	4.7 ± 2%	4.7 ± 2.3%	4.9 ± 2.2%	5.2 ± 2.2%	5.5 ± 2.2%
TELR22	TTAGGGTTAGGGTTAGGGTTArGGG	1.8 ± 1.3%	2.9 ± 1.8%	5.2 ± 3%	8.6 ± 2.9%	13.4 ± 4.4%	20.7 ± 4%
TELR23	TTAGGGTTAGGGTTAGGGTTAGrGG	1.4 ± 2.3%	1.4 ± 1.9%	2.1 ± 3%	1.1 ± 1.5%	1.1 ± 1.9%	1.2 ± 2%
TELR24	TTAGGGTTAGGGTTAGGGTTAGGrG	N.D.	N.D.	N.D.	N.D.	N.D.	N.D.

N.D. signifies not detected. The location of the ribonucleotide substitution is denoted with lower case r and underlined.

**Table 4.** Percentage of RNase H2 cleavage of G4s in the presence of sodium chloride

Sample	Sequence	1 min	5 min	15 min	30 min	60 min	120 min
dsTELR10	TTAGGGTTArGGGTTAGGGTTAGGG	97%	98%	98%	99%	99%	99%
TELR3	TTrAGGGTTAGGGTTAGGGTTAGGG	5.3 ± 8.2%	9.4 ± 4.6%	21.4 ± 2.6%	29.2 ± 2.3%	42.9 ± 1%	58.5 ± 0.6%
TELR4	TTArGGGTTAGGGTTAGGGTTAGGG	7.2 ± 6.4%	10.4 ± 9%	18.1 ± 4%	26.2 ± 2.7%	39.5 ± 1.4%	54.1 ± 5.2%
TELR5	TTAGrGGTTAGGGTTAGGGTTAGGG	N.D.	N.D.	N.D.	N.D.	N.D.	N.D.
TELR6	TTAGGrGTTAGGGTTAGGGTTAGGG	N.D.	N.D.	N.D.	N.D.	N.D.	N.D.
TELR9	TTAGGGTTrAGGGTTAGGGTTAGGG	13.5 ± 5.7%	15.5 ± 6%	20.6 ± 6.4%	25.7 ± 5.5%	33.5 ± 4.5%	42.1 ± 4.4%
TELR10	TTAGGGTTArGGGTTAGGGTTAGGG	7.4 ± 3.9%	20.9 ± 3%	43.6 ± 1.4%	63.4 ± 1%	81.4 ± 3.3%	92.6 ± 2.9%
TELR11	TTAGGGTTAGrGGTTAGGGTTAGGG	3.8 ± 5.2%	3.5 ± 4.4%	3.8 ± 4.3%	12.9 ± 12.8%	10.6 ± 9.4%	10.3 ± 7.9%
TELR12	TTAGGGTTAGGrGTTAGGGTTAGGG	7.5 ± 6.6%	0.3	0.6	0.6	9 ± 7.2%	6.6 ± 4.6%
TELR15	TTAGGGTTAGGGTTrAGGGTTAGGG	14.8 ± 6.5%	19.7 ± 5.1%	30.9 ± 3.4%	44.7 ± 1.4%	61.2 ± 0.6%	75.7 ± 1.8%
TELR16	TTAGGGTTAGGGTTArGGGTTAGGG	4.4 ± 3%	8.1 ± 2.9%	14.6 ± 3.2%	19.4 ± 3.7%	26.6 ± 3.8%	38.4 ± 5.8%
TELR17	TTAGGGTTAGGGTTAGrGGTTAGGG	3.3 ± 3.7%	3.8 ± 4.1%	3.8 ± 3.6%	4.7 ± 3.6%	6.5 ± 3.3%	9 ± 3.2%
TELR18	TTAGGGTTAGGGTTAGGrGTTAGGG	11.1 ± 9.6%				13.3 ± 12.4%	9.3 ± 8.1%
TELR21	TTAGGGTTAGGGTTAGGGTTrAGGG	13.4 ± 10.4%	16.7 ± 10.4%	12.6 ± 5.6%	15.7 ± 5.1%	20.7 ± 5.6%	22.9 ± 12.9%
TELR22	TTAGGGTTAGGGTTAGGGTTArGGG	12.4 ± 3.9%	51.4 ± 1.5%	87.9 ± 4.1%	99.3 ± 0.6%	99.9 ± 0.1%	99.3 ± 1.1%
TELR23	TTAGGGTTAGGGTTAGGGTTAGrGG	N.D.	N.D.	N.D.	N.D.	N.D.	N.D.
TELR24	TTAGGGTTAGGGTTAGGGTTAGGrG	N.D.	N.D.	N.D.	N.D.	N.D.	N.D.

N.D. signifies not detected. The location of the ribonucleotide substitution is denoted with lower case r and underlined.

### Circular dichroism

All CD experiments were performed using a Jasco-720 spectropolarimeter equipped with a thermocontroller a 2 mm quartz cuvette. For CD spectra generation, we performed 10 CD scans collected in the annealing buffer or kinetics buffer (100 mM NaCl, 50 mM Tris) at room temperature (23°C) from 220 to 340 nm with 1 nm data pitch, 2 nm bandwidth, 0.5 s response, and a scanning speed of 100 nm/min. The DNA sample was then loaded into the same cuvette for a final DNA concentration of 100 μM and scanned with the same settings. CD data were processed by subtracting the buffer spectra from the sample spectra. We performed CD melt by first identifying the wavelength at which a sample displayed the maximum ellipticity, and then by monitoring the ellipticity at that wavelength while increasing the temperature from 25°C to 95°C at a rate of 1°C/min. A reverse scan was then performed by monitoring the ellipticity at the same wavelength while decreasing the temperature from 95°C to 25°C at a rate of 1°C/min to reanneal the G4. Afterwards, a melting/reannealing spectral scan was performed to confirm that the original conformation was reformed. Melting points were calculated from the melting and cooling curves by generating a third-order polynomial for each experiment, taking the second derivative, and determining the root. Each experiment was done in triplicate (CD spectrum, melt curve, reanneal curve, and post-melt/reanneal curve spectrum).

### Single-molecule Förster Resonance Energy Transfer

All fluorescently labeled oligonucleotides were purchased from IDT. All the sample preparation and single-molecule Förster Resonance Energy Transfer (smFRET) experiments were conducted as reported earlier [46, 70]. Each duplex oligo was annealed by mixing 9.5 µM of the Cy5-labeled biotin strand and 10.5 µM of the Cy3 strands in 10 mM Tris–HCl, 100 mM KCl at pH 7.5, as reported earlier. The mixtures were incubated at 95°C for 2 min then slowly cooled to 37°C at a rate of 2°C per minute, and then cooled to room temperature at a rate of 5°C per minute. The single-molecule experiments were performed with a prism-type Total Internal Reflection Fluorescence microscope at room temperature (25 ± 2°C). Solid state 532 and 637nm OBIS lasers (Coherent, Saxonburg, PA) were used for excitation. The PEGlyted surface of slides and coverslips were prepared with the standard protocol and a mixture of m-PEG-5000 and biotin-PEG-5000 in mass ratio 40:1. All DNA samples were immobilized onto PEGylated surface via biotin−neutravidin interaction, and the data were collected in an imaging buffer (10 mM Tris, 100 mM KCl, 0.5% v/v D-glucose, 1 mg/ml glucose oxidase, 10 mM Trolox, and 2 μg/ml catalase) that generated an oxygen scavenging system. It is noteworthy that a 10 mM Trolox solution was prepared with 10 mM NaOH to enhance the solubility of Trolox. Data were acquired with an EMCCD camera at a 100 ms per frame rate and further processed using IDL and MATLAB codes. FRET efficiency (E) was calculated according to the following equation:


\begin{eqnarray*}
E = \frac{{F_A^D}}{{F_A^D + F_D^D}}.
\end{eqnarray*}

Where, $F_A^D$ is the acceptor (A) signal upon the donor (D) excitation; $F_D^D$ is the direct excitation signal of the donor [70]. Each FRET histogram was generated from 20 individual movies for 2 s long, which contained a total of 6000 or more individual molecules of a single Cy3–Cy5 pair. The trace counting results were generated from 900 or more smFRET traces for each oligo recorded in five or more experimental repeats that have both Cy3 and Cy5 signals throughout the 3-min record time. The traces in each movie were manually categorized into three different types of molecular behavior.

### Human RNase H2 expression and purification

The heterotrimeric human recombinant RNase H2 enzyme was expressed from the pET-hH2ABC plasmid expressed in the MIC1066 *Escherichia coli* strain, both of which were generously provided by R.J. Crouch (National Institute of Child Health and Human Development, Bethesda, MD, USA). The plasmid was transformed into the MIC1066 *E. coli* strain by heat shock. Transformed cells were grown at 37°C until an OD_600_ of ~0.7 was reached. RNase H2 expression was induced with 0.5 mM isopropyl β-d-1-thiogalactopyranoside (IPTG) at 20°C overnight (16 h). Cell pellets were frozen and stored at −80°C. Cells were lysed via sonication in a buffer containing 50 mM HEPES, pH 8.0, 300 mM NaCl, 20 mM imidazole, 1 mM Dithiothreitol (DTT), and a mixture of protease inhibitors (AEBSF, leupeptin, benzamidine, and pepstatin A). Lysate was cleared at 24 000 × *g* for 1 h. Cleared lysate was loaded onto a GE HIS column. RNase H2 was eluted with a 20–500 mM step imidazole gradient. Fractions containing RNase H2 were pooled and diluted to 50 mM HEPES, pH 8.0, 150 mM NaCl, and 250 mM imidazole. The sample was placed in Heparin buffer (50 mM HEPES, pH 8.0, 150 mM NaCl, and 1 mM DTT). The sample was then loaded onto a GE Heparin column and equilibrated with Heparin buffer. The protein was eluted with a 150 mM-2 M NaCl step gradient. Fractions were pooled, and RNase H2 was further purified by gel filtration using a GE Sephacryl column in a buffer containing 50 mM HEPES, pH 8.0, 150 mM NaCl, 1 mM ethylenediaminetetraacetic acid (EDTA), and 1 mM DTT. RNase H2 concentration was determined by absorbance at 280 nm using a NanoDrop (Thermo Fisher Scientific). The purified RNase H2 was then flash frozen in liquid nitrogen and stored at −80°C at a concentration of 8.71 mg/ml (100.91 uM).

### Human RNase H2 activity assays

The RNase H2 cleavage assay was performed with 5′ fluorescein (FAM)-labeled oligonucleotides. The reactions were performed using 400 nM RNase H2 and 50 nM DNA substrate in sodium reaction buffer [50 mM Tris, pH 8.0, 0.1 mg/ml bovine serum albumin (BSA), 1 mM DTT, 1% glycerol, and 25 mM NaCl] or potassium reaction buffer (50 mM HEPES, pH 8.0, 0.1 mg/ml BSA, 1 mM DTT, 1% glycerol, and 25 mM KCl). Reactions were initiated by the addition of 10 mM MgCl_2_. Aliquots were taken at the specified time points, and the reactions were quenched using a loading dye containing 78% formamide, 100 mM EDTA, 0.25 mg/ml bromophenol blue, and 0.25 mg/ml xylene cyanol. Samples were then incubated at 95°C for 5 min and ran on a 22% denaturing polyacrylamide gel (CBS). Reaction products were visualized using a GE Amersham Typhoon. Gels were analyzed using ImageQuant TL v8.1.0.0. To calculate the percentage of sample that was cleaved by RNase H2, we used ImageQuant to quantify the amount of DNA in both the cleaved and uncleaved bands. Reactions were performed in triplicate, except for the dsTELR-10 reactions, which were performed to confirm the activity of RNase H2. Percent cleavage was calculated by dividing the amount of DNA that was cleaved by the total DNA content (i.e. the sum of the cleaved DNA and uncleaved DNA).

## Results

### Determination of the impact of rNMP on G4 conformation and stability

**Figure 2.**
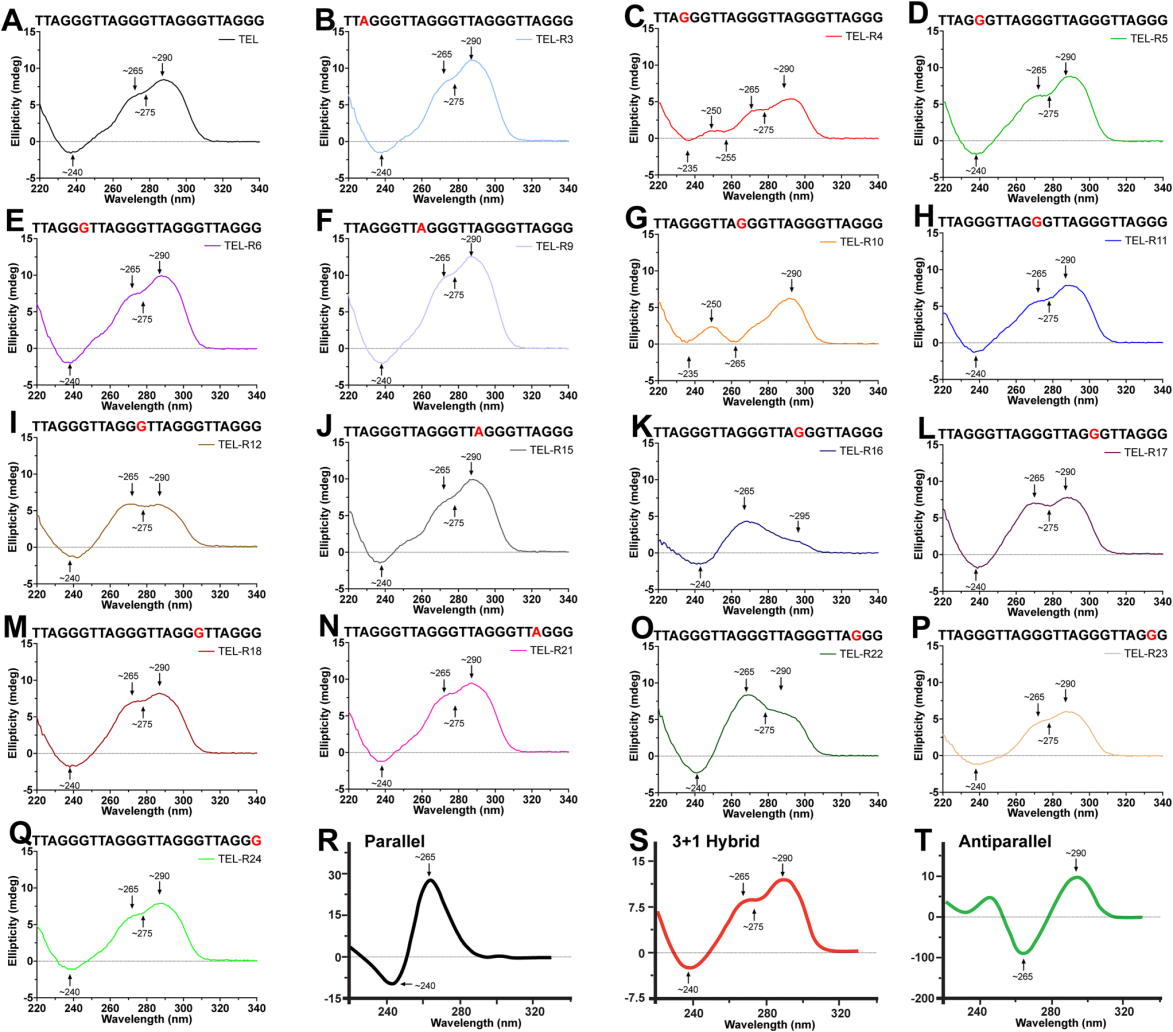
CD characterization of TEL sequences with rNMP substitutions. The sequence tested is listed above each panel (**A**–**Q**) and corresponds to an average of 10 CD scans performed at 23°C from 220 to 340 nm, in 100 mM KCl and 50 mM HEPES, with oligos at a concentration of 100 μM. Red base denotes the rNMP substitution in each variant sequence. Panels **R–T** show representative CD spectra of parallel, antiparallel, and hybrid G4 (adapted from [28] with permission). The location of key maximum and minimum are indicated for each panel. Also see Table 1 for the conformation of each G4.

We first characterized the structural effects of rNMP substitution into single-stranded telomeric DNA. To do so, we took the minimal G4-forming human telomere sequence (4 TTAGGG repeats, (TTAGGG)_4_), and systematically substituted an rNMP in place of its dNMP equivalent (e.g. rGMP for dGMP) [37]. This generated 16 sequence variants, each with a single rNMP substitution. The sequence variants were named based on the position of the rNMP within the telomere sequence (Table 1). Next, we determined the primary G4 conformations adopted by each sequence variant using circular dichroism (CD) (Fig. 2). CD has been extensively used to interrogate the conformations of G4s based on their local maxima and minima [65–68]. Parallel G4s have a maximum at ∼265 nm and a minimum at ∼240 nm, antiparallel chair G4s have a maximum at ∼290 nm and a minimum at ∼265 nm, antiparallel basket G4s have maxima at ∼250 and ∼290 nm and minima at ∼235 nm, and ∼265 nm, and 3 + 1 parallel/antiparallel G4s have maxima at ∼265 and ∼290 nm and minima at ∼240 and ∼275 nm [65–68].

Using CD, we observed that the canonical pure DNA oligo, TEL, has a spectrum consistent with a 3 + 1 parallel/antiparallel G4 conformation with a maxima at 265 and 290 nm and minima at 240 and 275 nm (Fig. 2A and Table 1), consistent with prior *in vitro* results [37]. We next used CD to determine the spectrum of the sequence variants with an rNMP substitution based on relative shifts in the spectral features compared to established reference spectra for parallel, antiparallel, and hybrid G-quadruplexes (Fig. 2R, S, and T; Supplementary Fig. S1) [28, 65, 68, 69]. TELR-3, TELR-5, TELR-6, TELR-9, TELR-11, TELR-12, TELR-15, TELR-17, TELR-18, TELR-21, TELR-23, and TELR-24 also had spectra consistent with a 3 + 1 parallel/antiparallel G4 conformation (Fig. 2B, D, E, F, H, I, J, L, M, N, P, and Q; Table 1). Importantly, while the TELR-12 and TELR-17 spectra showed characteristics of a 3 + 1 parallel/antiparallel G4, it did not have distinctly defined peaks: instead, the spectra contained a somewhat diffuse plateau between 260 and 290 nm, which may indicate that not all of the oligonucleotide formed a G4 (Fig. 2I and L) [68]. When considering the general trend, these results indicate that rNMP substitutions at these positions did not significantly alter the G4 confirmation. We also found that an rA substitution within the loop linker region between tetrads did not alter conformation when compared to DNA only TEL sequence (Fig. 2B, F, J, and N; Table 1). In contrast, rNMP substitution in TEL-R4, TEL-R10, TEL-R16, and TEL-R22 induced conformational transitions in the resulting G4 structures, exhibiting CD profiles indicative of conformational heterogeneity (Fig. 2C, G, K, and O; Table 1). Interestingly, each of these substitutions with an altered G4 conformation had a rNMP substitution located at the first G (G1) in each of the four telomeric repeats (i.e. TTAGGG). In the case of TEL-R4, the spectrum was consistent with both an antiparallel basket and a 3 + 1 parallel/antiparallel, indicating that TEL-R4 formed a mixed population of antiparallel basket and 3 + 1 parallel/antiparallel G4s (Fig. 2C and Table 1). For TEL-R10, the spectrum was consistent with an antiparallel basket G4 (Fig. 2G and Table 1) and an intermolecular G4, which has a positive peak at 250 nm and no significant positive peak at 275 nm. For TEL-R16, the spectrum was consistent with a mixed population of parallel and antiparallel chair G4s (Fig. 2K and Table 1). For TEL-R22, the spectrum was consistent with a mixed population of 3 + 1 parallel/antiparallel and parallel G4s (Fig. 2O and Table 1). Collectively, these results indicate that the first dGMP in a repeat is a critical position within a G4-forming sequence that, when substituted with a rGMP, alters G4 conformational dynamics.

**Figure 3.**
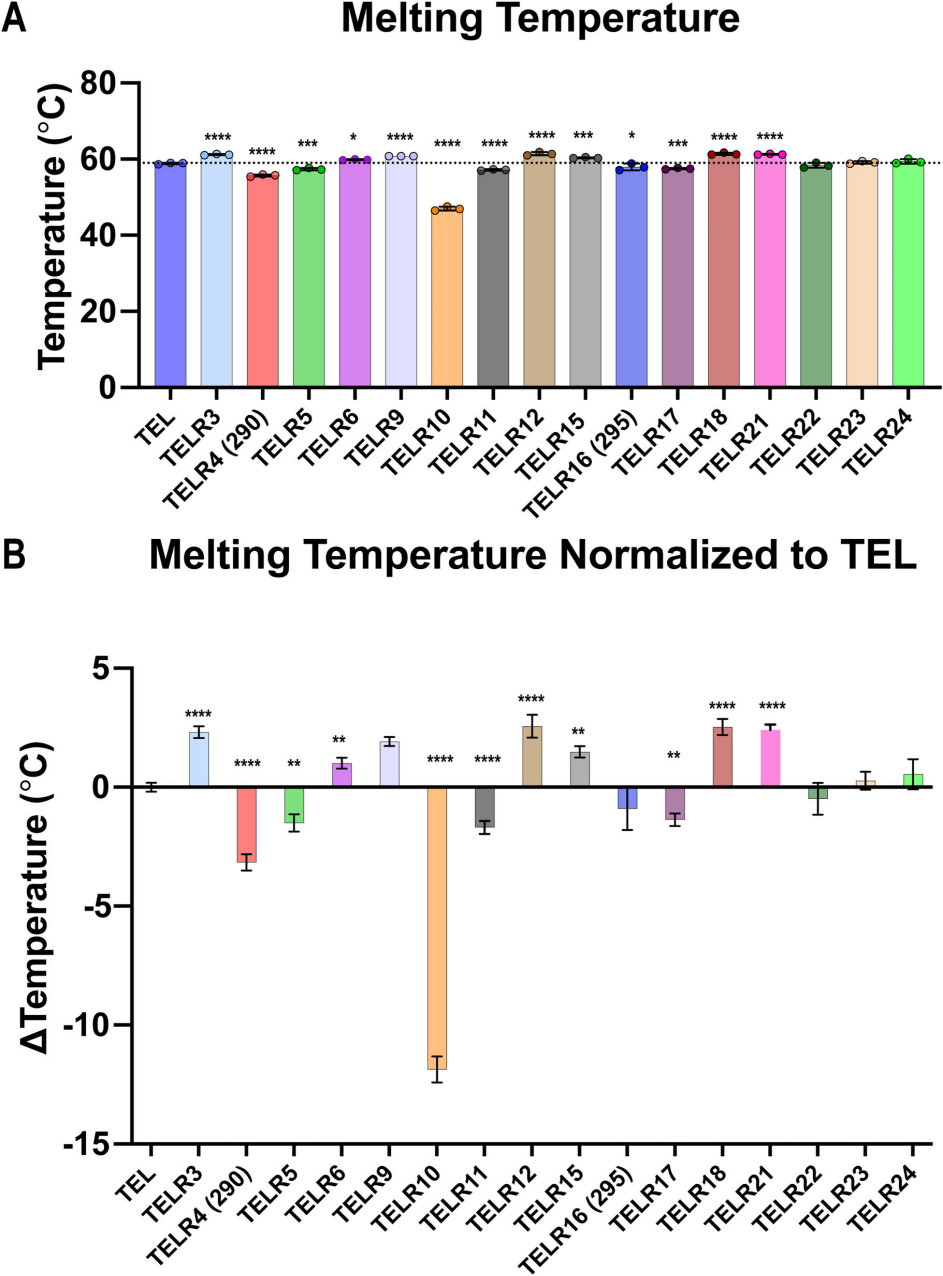
Characterization of thermal stability of TEL sequences with rNMP substitutions. (**A**) The melting point for each CD spectrum (*N* = 3) was calculated, and the average of three runs is shown with the data points indicated, error bars represent standard deviation. Dashed line at 58.86 is the melting temperature of the canonical TEL oligo of only DNA. (**B**) Melting temperatures normalized to TEL is shown to visualize the change in temperature for each sequence following rNMP insertion. The statistical analysis was performed by GraphPad Prism 10.0.2 using one-way analysis of variance with *P < .05, **P < .001, ***P < .001, ****P < .0001).

Given the potential disruptive effects of the additional hydroxyl group in the rNMP, we decided to investigate the stability of each sequence variant by quantifying its melting temperature, as well as its reannealing temperature. To accomplish this, we used the wavelength at which each sequence variant displayed the greatest ellipticity from our CD spectral analysis (Fig. 2) while increasing the temperature from 25°C to 95°C, followed by returning the temperature to 25°C, as has been done previously [65]. For the sequence variants that formed mixed populations, we chose the wavelengths with the strongest ellipticity that were specific to each conformation and performed sequential cycles of denaturation and reannealing: this allowed us to determine the thermal stability of each G4 conformation within a sample. To ensure that we analyzed true melting curves, we performed a post-denaturation and reannealing spectrum scan on each of the sequence variants to confirm that the original G4 structure reformed after cooling the sample from 95°C to 25°C (Supplementary Fig. S1). All post-denaturation and reannealing spectrum scans were similar, demonstrating that each of the sequence variants reformed into a conformation like their original conformation(s). In general, we observed that rNMP substitution induced relatively small, yet statistically significant, changes in the thermal stability of many of the sequence variants (Fig. 3A and Table 1). However, TEL-R10, which contains a G1 rNMP substitution, underwent a large reduction in its thermal stability compared to TEL, from 58.9 ± 0.2°C to 47.0 ± 0.5°C for melting temperature (Fig. 3 and Table 1). When comparing melting temperatures normalized to TEL, we identified three general trends with a few exceptions that are primarily in the terminal repeat (Fig. 3B and Table 1). First, substitution of the first or second dG in a repeat with an rG generally reduced thermal stability, except in TEL-R16, TEL-R22, and TEL-R23. Second, substitution of the third dG in a repeat with an rG increased stability, with TEL-R24 as the sole exception. Third, substituting a dA in the linker region between G tetrads with an rA increased thermal stability. These same trends were observed when analyzing the reannealing temperatures for the oligos (Supplementary Fig. 2A). Additionally, when normalized to TEL, the reannealing temperatures show the same three general trends as the melting temperatures (Supplementary Fig. 2B).

### Determination of the impact of rNMP on G4 conformational dynamics

**Figure 4.**
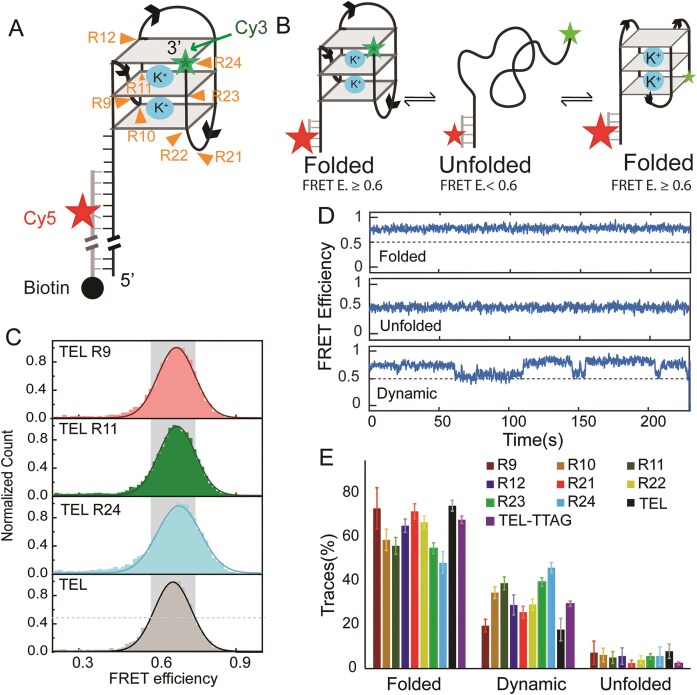
smFRET revealed increases in dynamic refolding due to rNMP insertion. (**A**) G4 structure formed at the 3′ end of the single-stranded (ss) overhang region of telomeric DNA, with an 18-nt long biotinylated duplex region at the 5′ end. The biotinylated portion is tethered to the PEGylated slide via the Biotin–Neutravidin interaction. (**B**) The change of G4 conformations revealed by changes in FRET efficiency. (**C**) smFRET histogram of selected constructs. The high-FRET value (gray) indicates folded population. TEL is used as positive control. (**D**) Real-time smFRET trajectories of different types of molecular behavior. Folded static traces show constant FRET *E* values between 0.6 and 0.9, unfolded traces show values below 0.6, and dynamic traces exhibit FRET E. value jumps from 0.6 to 0.9 in real-time. (**E**) Quantitative analysis of different types of telomeric constructs. TEL and TEL TTAG are DNA only telomere constructs. The error bars represent standard deviation for *n* = 5 repetitions. In total, 954 ± 365 good traces were analyzed for each construct.

To investigate the effect of rNMP substitution at different positions of the telomeric G4, we used smFRET to study eight rNMP-substituted telomeric sequences. These sites were chosen to capture trends across G1–G3 positions, assess loop contributions to G4 dynamics, investigate the second repeat due to the melting shift observed for TEL-R10 (Fig. 3), and included the terminal repeat, given prior studies looking at DNA damage placed it in this repeat, which provides additional comparison to other forms of DNA damage [45]. All DNA constructs share an 18-base pairs (bp) nontelomeric double-stranded (dsDNA) region and a 24 nucleotide (nt) single-stranded telomeric overhang, and a single rNMP substitution (Fig. 4A and Supplementary Table 1). Each DNA sample has two fluorescent dyes, Cy3 at the 3′ end of the single-stranded overhang and Cy5 at the complementary strand of the dsDNA nontelemetric dsDNA region (Supplementary Table 1). The dsDNA handle has the Cy5 located between the fourth and fifth bases from the dsDNA–ssDNA junction, which has been used for studying G4 conformational dynamics with smFRET [70, 71]. When this G4 undergoes refolding or conformational change, the distance between the two dyes changes as the Cy3 moves away or toward the Cy5 fixed in the handle. This distance change can be recorded as FRET efficiency (FRET E.) changes. The collective change of distances can be identified by the shift of smFRET histogram, and the real-time distance fluctuation will give dynamic smFRET trajectory at a single spot (Fig. 4B).

The smFRET histogram of all tested rNMP constructs in 100 mM KCl showed a similar single high FRET E. peak centered at 0.67 ± 0.01, which remains consistent with different repetitions (Fig. 4C and Supplementary Fig. 3). This smFRET peak center is the same as the smFRET peak center (0.67 ± 0.01) of canonical pure DNA oligo, TEL, which is fully folded into a G4 under this experimental condition [70]. The similar FRET values of all the constructs indicate majority of the oligos fold into G4 with the 5′ to 3′ distance (see Fig. 4C, Supplementary Fig. 3, and Table 2). Among all the rNMP substituted oligos, TEL-R24 has a slightly broader peak than TEL (see Table 2), which indicates multiple conformations or conformational dynamics co-exist in this oligo.

The conformational dynamics of each oligo is elucidated by real-time smFRET traces of each rNMP substituted and the canonical telomeric DNA (TEL). As demonstrated in Fig. 4D, three types of smFRET traces were observed in each construct, as we reported earlier for telomeric G4. The first type includes all the molecules that have a FRET E. equal to or higher than 0.60 throughout the 3-min record time, which is considered static-folded. The second type includes all the molecules that remain at a FRET E. below 0.6 throughout the 3-min record time, which is considered unfolded. The rest of the molecules, which undergo one or more states of FRET E., are considered as dynamic. The 0.6 FRET E. cutoff of folded G4 was chosen based on our earlier smFRET data of TEL in 10 mM Tris–HCl, which has a histogram centered at 0.4 ± 0.05 FRET E. and the full range of the population spread up to FRET 0.6 [70]. Counting a total of 900 traces or more of each oligo for the number of traces in each type gives a different percentage of molecular behavior. In general, each construct with an rGMP substitution has more dynamic traces than regular TEL DNA-only sequence, but the ones with rAMP substitution do not. (Fig. 4E and Table 2). Substitution of rGMP in the TEL_sm_ at the G1 position in different repeats resulted in an increase in dynamic traces, with TEL-R10 at 34.6 ± 4.9%, TEL-R22 at 29.2 ± 2.8%, compared to TEL_sm_ at 17.8 ± 5.1. Substitutions of rGMP at the G2 position had a similar effect of increasing dynamic traces, compared to TEL_sm_, with TEL-R11 having 38.9 ± 3.0% and TEL-R23 having 39.6 ± 2.0%. All other rNMP substitutions in the loop or G3 position have different effects on trace count, as discussed below.

The oligos with rAMP substitution in the loops, TEL-R9 and TEL-R21, showed a similar percentage of static-folded traces (73.1 ± 9.43% and 71.8 ± 3.45%, respectively) as TEL (74.3 ± 2.50%) (Fig. 4E and Table 2), which depicted that rNMP insertion in the loop region of telomeric G4 does not change the stability of the G4 structure. The increase in their thermal stability is reflected in the percentage of dynamic and unfolded traces. The percentage of dynamic traces for TEL-R9 shows almost the same value (19.6 ± 2.9%) as TEL_sm_ (17.8 ± 5.1%). This similarity indicates that rNMP insertion in the first loop has less effect on the stability of G4. On the other hand, the slight increase of the dynamic traces (25.8 ± 2.71%) of TEL-R21 in comparison with TEL comes from the decline in the unfolded population of TEL-R21 (2.5 ± 1.4% versus 7.9 ± 3.3% for TEL) (Table 2). This indicates that TEL-R21 tends to fold into G4, and some of the unfolded population in TEL spent partial time at folded state, which makes the FRET traces fluctuate from folded to unfolded states rather than staying in a random-coil state. The reduction in unfolded and increase in dynamic behavior is consistent with the higher thermal stability of TEL-R21 (*T*_*m*_ = 61.26 ± 0.13°C) than TEL-R9 (*T*_*m*_ = 60.77 ± 0.02°C) and TEL (*T*_*m*_ = 58.86 ± 0.19°C). The difference between TEL-R9 and TEL-R21 can be explained by the orientation of the first and third loops and the stacking of the loop bases to the G4 core. In the 3 + 1 hybrid structure (PDB 2HY9), the first loop has a C5 to C5 distance of 0.9 nm and for the third loop the distance is 1.2 nm (Supplementary Fig. S4) [72]. The A bases in the third loop aligns with the guanine in the G4 core, which suggests a pi–pi interaction between the bases, while the A base in the first loop does not. The OH group of rAMP in the third loop may induce a sugar puckering that encourages this pi–pi interaction and help the adenine stack to the G4 core, resulting in less unfolded molecules and more dynamic molecules.

The oligo with rGMP substitution at the G1 position, TEL-R10 and TEL-R22, showed a lower percentage of static folded traces (58.8 ± 4.9% and 66.8 ± 2.8%, respectively) and a higher percentage of dynamic traces (34.6 ± 4.9% and 29.2 ± 2.8%, respectively) than the rAMP-substituted oligo, as shown in Table 2. Their lower static-folded traces and higher dynamic traces than TEL indicate that more molecules adopt a looser conformation that is capable of refolding. The formation of additional hydrogen bonds between ribose and the loop bases mentioned earlier may contribute to the stabilization of alternative conformations, encourage G4 refolding, and reduce the overall stability.

Among the three rGMP substituted constructs in the second TTAGGG repeat, the construct with G2 substitution (TEL-R11) shows a higher percentage of dynamic traces (38.9 ± 3.0%) than the construct with G3 substitution (TEL-R12, 29.0 ± 4.6%), and the G1 construct is in between (34.6 ± 4.9%). It is interesting that the percentage of dynamic traces of three rGMP-substituted oligo in the second TTAGGG repeat has the order of TEL-R12 ≤ TEL-R10 ≤ TEL-R11. While the TEL-R12 has a similar percentage of dynamic traces as TEL-R10, and TEL-R10 has a similar percentage of dynamic traces as TEL-R11, the difference between TEL-R12 and TEL-R11 is beyond the error range. This position-dependent increase in dynamic traces at G2 position follows a similar order as our earlier report on guanine base damage, which revealed that the central guanine damage has the strongest effect on destabilizing the G4 core and induces the highest amount of dynamic traces [45–47]. The increase of dynamic traces of TEL-R11 than TEL-R12 is consistent with their variation in melting temperature, which is supported by our earlier report on how base damage and mutation at the G2 position affect structure more by loosening it than G3 [47]. It is interesting that TEL-R10 has the lowest melting temperature among the three but does not have the highest percentage of dynamic traces. This difference in thermal stability and dynamics might be explained by the competition between ribose-base interaction and core destabilization due to sugar puckering, which may be worth further investigation.

In the fourth TTAGGG repeat, which located at the 3′ end of the sequence, the substitution of rGMP at the G2 and G3 positions shows a different effect compared to the constructs of G2 and G3 substitutions in the second repeat. The static-folded traces of TEL-R23 and TEL-R24 (54.9 ± 2.5% and 48.2 ± 4.9%, respectively) show a decreasing trend from TEL-R22 (66.8 ± 2.8%). In contrast, the dynamic traces of TEL-R23 and TEL-R24 show a gradual increase (39.6 ± 1.9% and 46.1 ± 2.4%, respectively) from TEL-R22 (29.2 ± 2.8%). The percentage of dynamic traces (the opposite of static folded traces) of these three rGMP substituted oligos has the order of TEL-R24 > TEL-R23 > TEL-R22. The dynamic traces of TEL-22 are comparable to the dynamics traces of TEL-TTAG, a control oligo with the same canonical DNA sequences as TEL but contains an extra TTAG tail in the overhang (Supplementary Table S1). TEL-TTAG has more dynamic traces than TEL, which demonstrates the flapping end increases conformational dynamics as reported earlier [71, 70]. The similarity between TEL-R22 and the gradual increase in dynamics traces of TEL-R23 and TEL-R24 suggests that the insertion of rGMP at the last TTAGGG repeat induces end flapping, which could introduce more smFRET signal dynamics to an unstable core but not significantly reduces the thermal stability of G4 (see Table 2 and Supplementary Fig. S4).

### RNase H2 recognition and cleavage of rNMPs within G4

**Figure 5.**
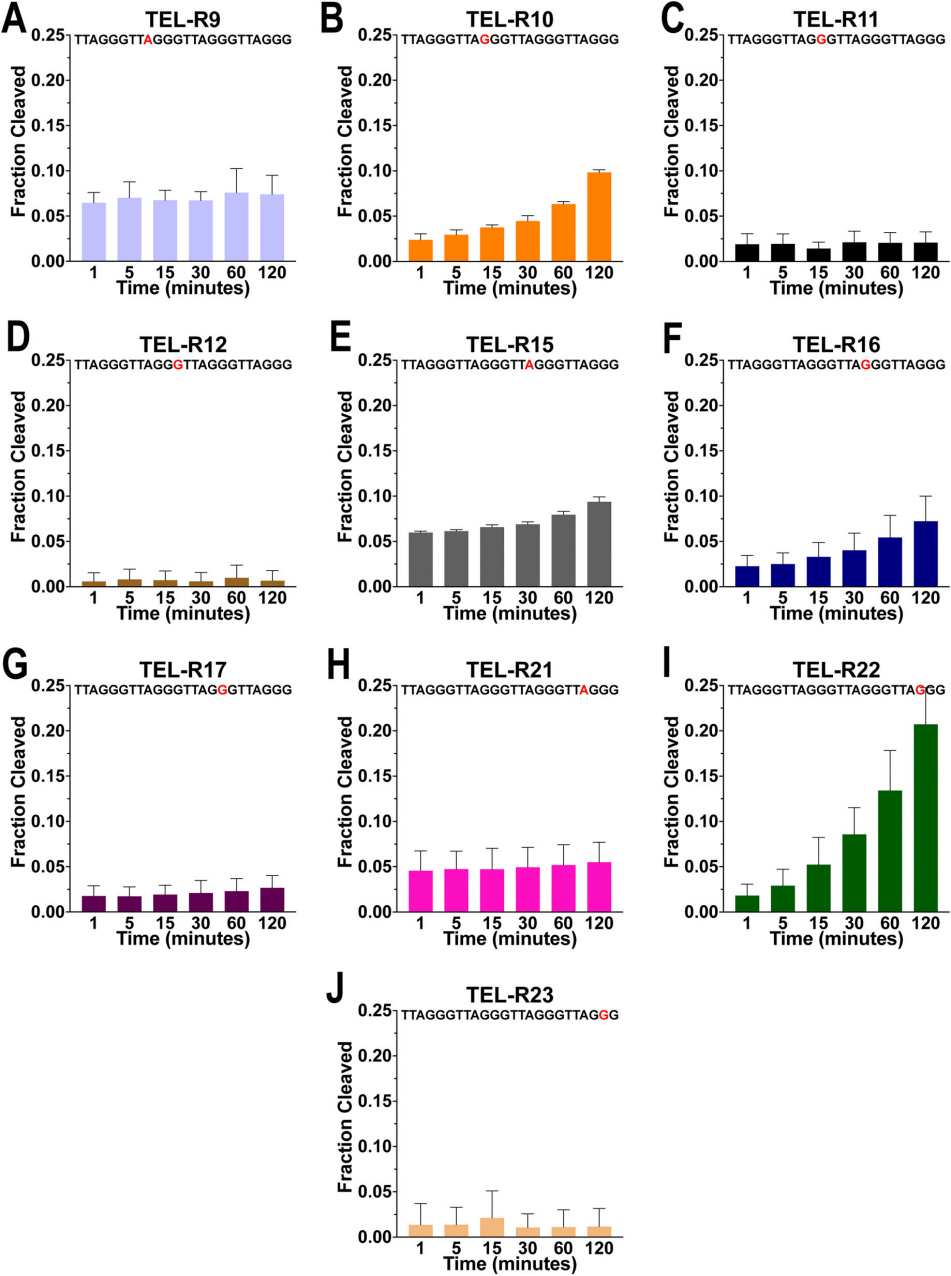
Cleavage of rNMPs within G4s by RNase H2 in the presence of potassium. Samples with the rNMP as the first G within a repeat (**B, F, I**) and those with the substitution in the loop (**A, E, H**) experienced the greatest cleavage. Those samples with the rNMP in the second or third position within a repeat had minimal, or no, cleavage (**C, D, G, J**). TEL-R3, TEL-R4, TEL-R5, TEL-R6, TEL-R18, and TEL-R24 are not displayed as the displayed non appreciable cleavage. The fraction cleaved is from three experiments, and the error bar represents standard deviation. Also see Table 3.

To repair rNMPs in DNA, RNase H2 must first recognize and cleave rNMP substitutions [58]. One biological context in which RNase H2 could encounter an rNMP is within a G4, as telomeric G4s can form when the G-rich strand becomes transiently single-stranded during replication, transcription, or telomerase extension [19, 73–76]. We therefore sought to determine whether RNase H2 has differential ability to recognize and cleave intramolecular telomeric rNMPs within G4s, as substrate recognition is a prerequisite for repair of rNMP insertion into DNA [58]. To do so, we treated each sequence variant with human recombinant RNase H2 for up to 2 h and measured cleavage. Because potassium is the most prevalent monovalent cation bound to G4s in cells, we began this characterization using reaction buffer containing 100 mM potassium chloride [77, 78]. We first confirmed that RNase H2 was able to cleave duplex telomeric DNA with a rNMP substitution, with 96% cleavage after 1 min (Table 3 and Supplementary Fig. S5). However, when the sequence variants were folded into a G4, RNase H2 was largely unable to cleave sequence variants that had rNMP substitutions of either the second or third dG in a repeat (G2 and G3, respectively) (Table 3). After 2 h of treatment with RNase H2, we detected no cleavage product for TEL-R3 (A), TEL-R4 (G1), TEL-R5 (G2), TEL-R6 (G3), TEL-R18 (G3), or TEL-R24 (G3) (Supplementary Figs S6–9 and S11). Similarly, we observed minimal cleavage for TEL-R11 (G2), TEL-R12 (G3), TEL-R17 (G2), and TEL-R23 (G2), which had 2.1%, 1.0%, 2.7%, and 1.2% cleavage, respectively (Table 3). In contrast, RNase H2 was able to induce small amounts of cleavage in sequence variants that had rNMP substitutions of either the first dG in a repeat (G1), or the dA (A) in the loops between G tetrads within the G4 (Fig. 5). After 2 h of treatment with RNase H2, we detected cleavage for TEL-R9 (A), TEL-R10 (G1), TEL-R15 (A), TEL-R16 (G1), TEL-R21 (A), and TEL-R22 (G1), which had 7.4%, 9.8%, 9.4%, 7.2%, 5.5%, and 20.7% cleavage, respectively (Table 3 and Supplementary Fig. S10). Interestingly, we noticed deviation from this trend for TEL-R3 and TEL-R4, which had an rNMP substitution in the loop A and G1, respectively; these instead exhibited no cleavage. The reason for this is not clear, but we hypothesize that the DNA-binding footprint of RNase H2 results in an inability to bind to the DNA at these positions. We conclude that the position of the rNMP substitution within the G4 may determine whether the substitution can be repaired efficiently.

**Figure 6.**
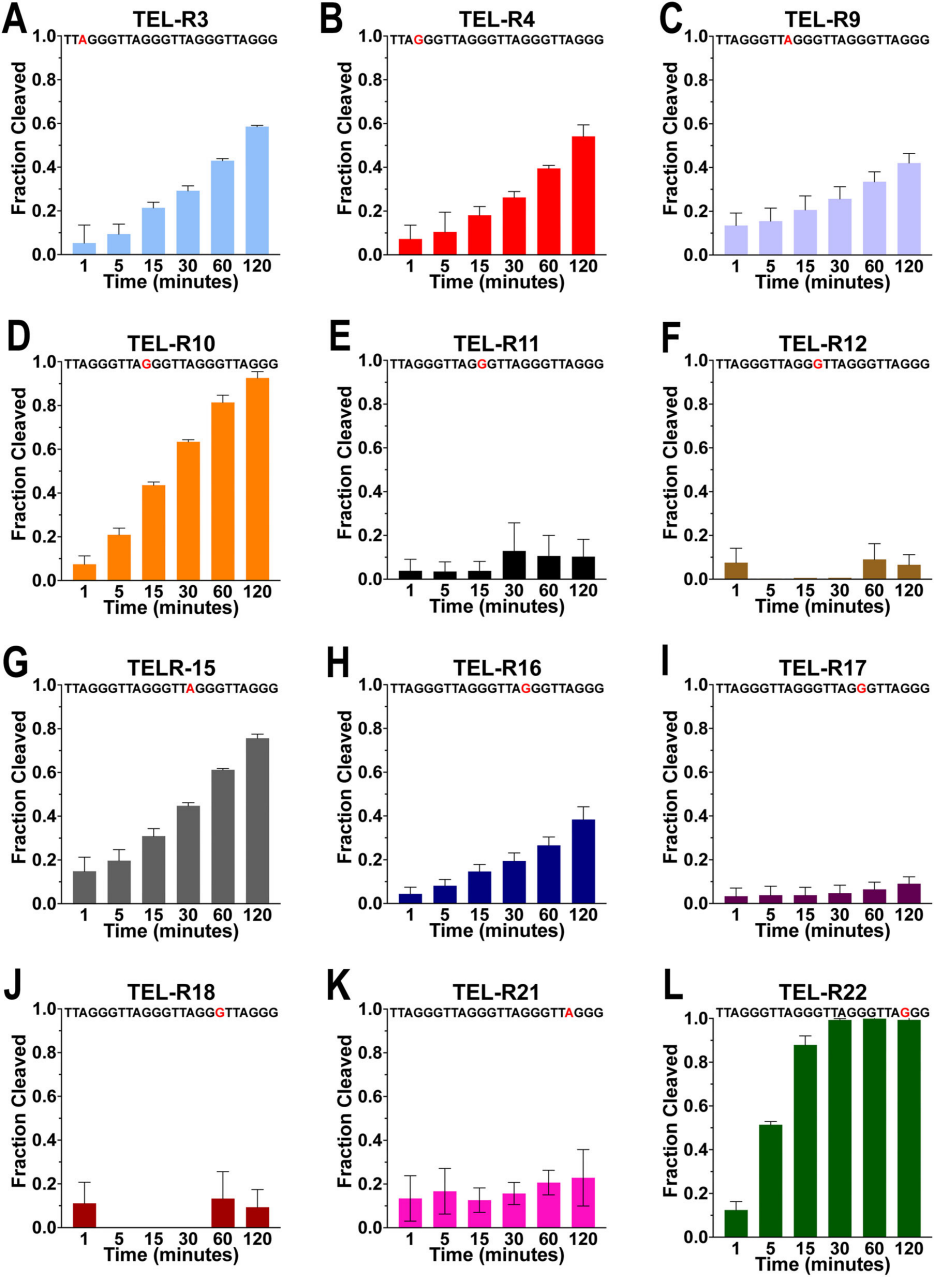
Cleavage of rNMPs within G4s by RNase H2 in the presence of sodium. Samples with the rNMP as the first G within a repeat (**B, D, H, L**) and those with the substitution in the loop (**A, C, G, K**) experienced the greatest cleavage. Those samples with the rNMP in the second or third position within a repeat had minimal, or no, cleavage (**E, F, I, J**). TEL-R5, TEL-R6, TEL-R23, and TELR-24 are not displayed as the displayed non appreciable cleavage. The fraction cleaved is from three experiments, and the error bar represents standard deviation. Also see Table 4.

We next investigated if the stability of the G4 altered the activity of RNase H2 cleavage. Stabilization of G4s requires a monovalent cation, and different monovalent cations provide different degrees of stabilization [79, 80]. To investigate if stability has an impact on repair, we repeated the RNase H2 cleavage assay as before, but in the presence of 100 mM sodium chloride rather than potassium chloride. G4s bound to sodium have reduced stability compared to G4s bound to potassium, due to sodium’s smaller atomic radius [79, 80]. We first confirmed that RNase H2 was able to cleave duplex telomeric DNA with a rNMP substitution in the presence of sodium, with 97% cleavage after 1 min (Table 4 and Supplementary Fig. S12). We found that, in general, sodium resulted in more frequent cleavage of rNMPs compared to potassium (Fig. 6 and Table 4). Similar to results in potassium, sequence variants with substitutions in G2 and G3 showed less cleavage than substitutions in G1 or A. After 2 h of treatment with RNase H2, TEL-R5 (G2), TEL-R6 (G3), TEL-R23 (G3), and TEL-R24 (G3) showed no activity, while TEL-R11 (G2), TEL-R12 (G3), TEL-R17 (G2), and TEL-R18 (G3) had 10.3%, 6.6%, 9.0%, and 9.3% cleavage, respectively (Table 4 and Supplementary Figs 14–16 and 18–21). Additionally, similar to results in potassium, sequence variants with the most frequent cleavage are those with rNMP substitutions for the first G of a repeat, or for an A in the linker region. TEL-R3 (A), TEL-R4 (G1), TEL-R9 (A), TEL-R10 (G1), TEL-R15 (A), TEL-R16 (G1), TEL-R21 (A), and TEL-R22 (G1) had 58.5%, 54.1%, 42.1%, 92.6%, 75.7%, 38.4%, 22.9%, and 99.3% cleavage, respectively (Table 4) (Supplementary Figs 13–18 and 21). Although the pattern of cleavage was similar in samples treated with sodium or potassium, sodium resulted in a higher percent cleavage compared to potassium. This indicates that the stability of the G4 is a factor in the ability of RNase H2 to cleave at an rNMP.

After concluding that cleavage of rNMPs in G4s is influenced by the cation in the buffer solution that stabilizes the tetrads, we decided to reexamine the CD spectra of the sequence variants in the presence of sodium. We performed CD spectra analysis as before, replacing potassium with sodium in the buffer solution. The CD spectra indicated that the presence of sodium altered the conformation of the sequence variants. Regardless of their initial conformation in potassium, each sequence variant adopted the antiparallel basket conformation in sodium (Supplementary Fig. S22). TEL-R4, TEL-R12, TEL-R16, TEL-R21, and TEL-R23 formed the antiparallel chair G4 in addition to the antiparallel basket G4 (Supplementary Fig. 22C, I, K, N, and P). These results underscore the importance of the conditions in which a G4 sequence forms, as the conditions can have a drastic impact on the conformation.

## Discussion

Here, we have taken the canonical human telomeric sequence (TTAGGG)_4_, and systematically investigated 16 variants in which a single dNMP was substituted with its rNMP equivalent. The canonical human telomeric sequence (TTAGGG)_4_ (TEL) forms a 3 + 1 parallel/antiparallel G4 conformation. In contrast, TEL-R4, TEL-R10, TEL-R16, and TEL-R22, sequence variants in which the first dG in a repeat was replaced with rG, formed different G4 conformations: TEL-R4 formed both antiparallel basket and 3 + 1 parallel/antiparallel, TEL-R10 formed antiparallel basket, TEL-R16 formed both parallel and antiparallel chair, and TEL-R22 formed both 3 + 1 parallel/antiparallel and parallel (Table 1). We observed that changes in conformation only occurred when the rNMP substitution occurred at the first dG position. This leads us to believe that that the first G in a repeat is a critical position, which has a great impact on the folding and conformation of the G4. It is likely that these results are generalizable to many G4-forming sequences and are not exclusive attributes of telomeric sequences.

Our thermal stability studies reveal a general trend: replacing the first or second dG in a repeat with an rG decreases thermal stability and increases conformational dynamics. TEL-R10, which formed an antiparallel basket G4 conformation, had the greatest decrease in thermal stability. This may be explained by the X-ray crystal structure of the antiparallel basket conformation, which reveals that the guanines within the tetrads are not completely planar with each other (Fig. 1). When the guanines are not planar, the hydrogen bonding is not at its strongest angle, thereby weakening the stability of the structure [81]. An alternative explanation is that TEL-R10 prefers to form intermolecular G4s that have lower melting temperature than intramolecular G4, which is worth studying in the future. For TEL-R4, the melting temperature at 275 nm (the wavelength most specific for the 3 + 1 parallel/antiparallel conformation) remained unchanged compared to TEL; in contrast, the melting temperature at 290 nm (the wavelength associated with both the 3 + 1 parallel/antiparallel and the basket conformations) significantly decreased. For TEL-R16 at 295 nm, we see a statistically significant decrease in melting temperature, but not for TEL-R16 at 270 nm, the wavelength specific for the 3 + 1 conformation. These data suggest that antiparallel conformations are less stable than parallel conformations, a finding consistent with existing literature [77]. For instance, increased loop length correlates with both decreased G4 stability and increased tendency to form antiparallel or hybrid conformations [66, 80, 82–89]. In the most extreme case, two single-nucleotide loops within a quadruplex-forming sequence will cause the formation of a parallel G4, regardless of the length of the remaining loop [80, 87]. This would indicate that antiparallel structures would tend to lower thermal stability.

Sequence variants with a substitution in the G2 position generally experienced a decrease in thermal stability, but not a change in conformation. Combining this result with the increased ratio of dynamic traces suggests that telomeres containing G2 rNMP substitution may fold into the same G4 as a canonical DNA telomere but are less able to remain stably folded. This is most likely due to the extra hydroxyl group of the rGMP perturbing the stable hydrogen bonds within the G4, indicating that the rNMPs can not only decrease the stability of G4s via changes in sterics, but can also decrease stability by perturbing hydrogen bonding within the G4. The overall conformation did not change severely because the two deoxyriboses at G1 and G2 keep the original preferred loop orientation. In contrast, sequence variants with substitutions in the G3 position, or in the loop region, experienced an increase in thermal stability. The increase in dynamic behavior cannot fully explain this stabilization effect, which suggests that conformation preference may overcome the destabilization effect of increase conformation dynamics. This interpretation is supported by the gradual increase of dynamic traces from the G2 to G3 positions in the last repeat, which is due to their flapping end but not a conformational change. Thus, the effects of rNMP substitution within G4s are complex and depend on the location of the substitution within the G4 forming sequence. The general destabilization effect of rGMP insertion in telomeric G4, and the preference of conformation or increase in structural dynamics, could be explained by the different sugar puckers of ribose versus deoxyribose. The solution NMR structure of the telomeric repeat-containing RNA (TERRA, rUUAGGG) G4 shows with *anti*-glycosidic bonds, in which the riboses adopt predominately C3′-endo configuration while DNA G4s have high polymorphism, and sugar puckering drives G4 refolding [90, 91]. It is also known that a single ribose substitution in dsDNA can drive the double helix conformation from B- to A-form [52]. Although it is uncertain whether the ribose in the hybrid G4 prefers the C3′-endo configuration over C2′-endo as they are in the hybrid double helix, the similar effect of hydroxy group on sugar pucker could also contribute to the G4 destabilization effect we observed.

We found that RNase H2 had difficulty recognizing and cleaving rNMPs in the context of a G4, an essential step for the initiation of the RER pathway. This was expected, as it has been previously shown that RNase H2 has difficulty cleaving rNMPs in DNA folded into some form of secondary structure [92]. However, in our study, we did see some cleavage of rNMPs within a G4. In the presence of potassium, we observed a small amount of cleavage that, in general, only occurred when either the rG was in the first position within a G repeat or when an rA was in the loop region. This is most likely due to accessibility of the ssDNA: when the rNMP is in these positions, RNase H2 is capable of accessing the lesion and initiating cleavage. RNase H2 binds to DNA 5′ of the rNMP to initiate its repair; thus, for an rG1 or an rA in the loop region, RNase H2 is not hindered by adjacent loops. However, when the rNMP is in other positions, adjacent loops do seem to hinder the ability of RNase H2 to cleave the DNA.

When G4s are in the presence of sodium, RNase H2 more frequently cleaves the rNMPs, but the pattern of cleavage with respect to the location of the rNMPs remains unchanged. Additionally, in the presence of sodium, all sequence variants formed either antiparallel basket G4 or antiparallel basket and antiparallel chair G4s. This conformational shift likely increases RNase H2’s access to the rNMP, explaining the higher frequency of cleavage in sodium. The conversion of the G4 conformation into an antiparallel conformation in sodium was not unexpected: it is well known that antiparallel conformations are favored in sodium, while parallel structures are favored in potassium [80, 86, 93]. This is thought to result from differences in these cations’ atomic radii leading to different effects on G4 stability. Larger potassium cations will be positioned between two tetrads and can thus coordinate with eight guanines, while smaller sodium cations will be positioned in the middle of a single tetrad. As such, sodium cations in the terminal quartets can interact with loop bases, which is why antiparallel conformations are more stable than parallel conformations in sodium [79, 80]. Future structural studies would help to confirm that RNase H2’s ability to initiate the repair of rNMPs in the context of G4s is dependent on its ability to access the lesion.

Within the cell, insertion of an rNMP can have deleterious consequences. When telomerase inserts an rNMP into the single-stranded telomeric overhang, the resulting effect will depend on where in the repeat the rNMP is inserted. If the first G in a repeat is substituted, the sequence is predisposed to form an antiparallel conformation, shifting the G4 population away from the typical conformation. If the second G in a repeat is substituted, the G4 will have decreased stability, even if the conformation is not altered. If the last G in a repeat, or a base in the loop, is substituted, the G4 will have increased stability. Furthermore, if the rNMP is substituted in a location inaccessible to RNase H2, the rNMP will likely remain unrepaired. It has been shown that antiparallel G4s inhibit telomerase [44, 94]. This inhibition of telomere extension is detrimental to replicating cells, and ligands that stabilize G4s have been explored as cancer therapeutics [95–97, 98]. Thus, rNMP insertion into telomeric sequences may alter G4s into conformations that will either inhibit telomerase or stabilize the G4 and inhibit extension of telomeres. Given the difficulty of repairing an rNMP lesion within a G4, these lesions, and their effects, could persist. All together, we show that insertion of rNMPs into telomeric sequences can have deleterious consequences for telomere integrity.

## Supplementary Material

gkaf1501_Supplemental_File

## Data Availability

The data underlying this article are available in the article and in its online supplementary material.
