# Supplementary material for: Effects of ribonucleotides on telomeric G4 formation, dynamics, and initiation of ribonucleotide excision repair by RNase H2
Source: Nucleic Acids Res. 2026 Jan 14;54(2):gkaf1501. doi: 10.1093/nar/gkaf1501 (PMC12802939; doi:10.1093/nar/gkaf1501)
Supplement: gkaf1501_Supplemental_File [file gkaf1501_supplemental_file.docx]

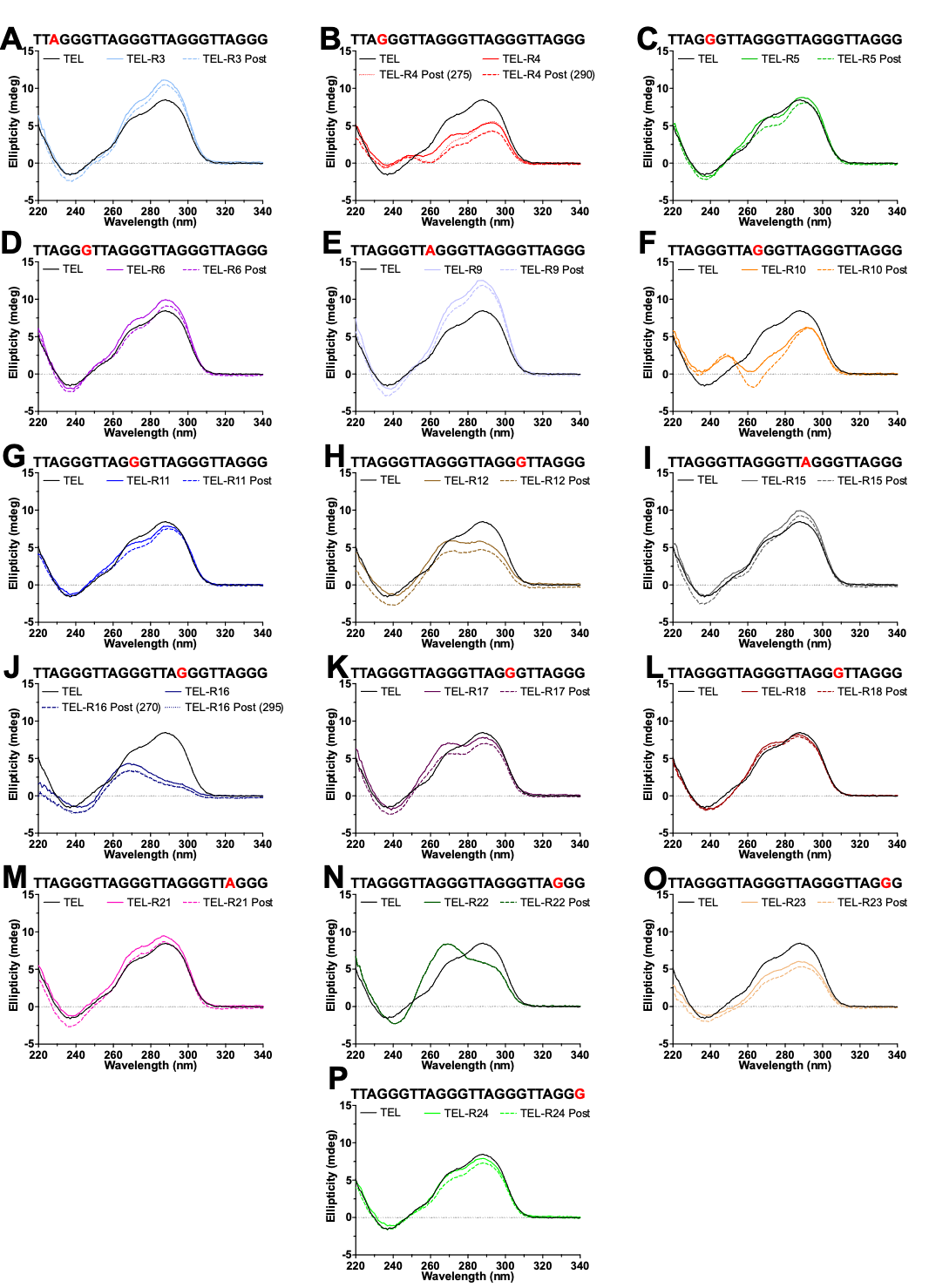


Supplemental Figure 1. Circular Dichroism characterization of TEL sequences. (**A-P**) 10 CD scans performed at 23°C from 220 nm to 340 nm in 100 mM KCl and 50 mM HEPES with oligos at a concentration of 100 μM. Black curve in each panel is the spectrum of TEL with minima at ~275 nm, ~245 nm and maxima at ~290 nm, ~265 nm indicative of a 3+1 G4 conformation. Solid curve in each panel denotes the spectrum for each sample. Dashed lines represent the spectrum after melting and reannealing oligo. (**A, C, D, E, G, I, K, L, M, O, P**) Spectra consistent with 3+1 G4 conformation. (**B**) TEL-R4: spectrum consistent with a mixed population of antiparallel basket and 3+1 G4 conformations. (**F**) TEL-R10: spectrum consistent with an antiparallel basket G4 conformation. (**J**) TEL-R16: spectrum consistent with a mixed population of parallel and antiparallel chair G4 conformations. (**N**) TEL-R22: spectrum consistent with a mixed population of 3+1 and parallel G4 conformations. (**H**) TEL-R12: spectrum consistent with weak formation of 3+1 G4 conformations.


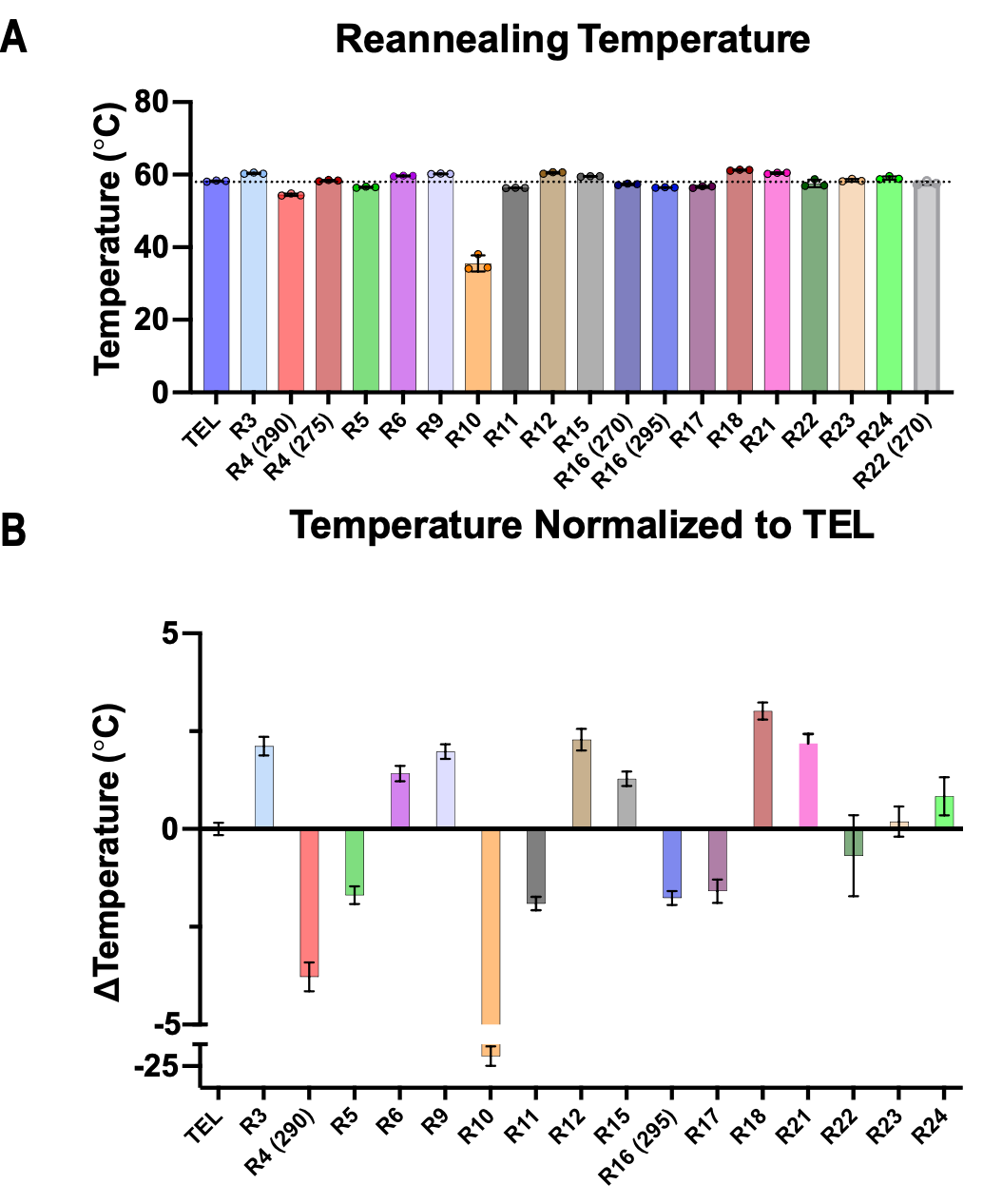


Supplemental Figure 2. Characterization of thermal stability of TEL sequences. CD signal was monitored at the wavelength at which each oligo displayed the strongest ellipticity (290 nm for all except TEL-R16 which was monitored at 295 nm), while decreasing the temperature from 95 °C to 25 °C at a rate of 1 °C every minute to look for the emergence of the G4 signal. N=3 (**A**) reannealing points were calculated by generating a third order polynomial for each run and taking the second derivative and determining the root. Results are the average of three runs. Dashed line at 58.22 is the melting temperature of the canonical TEL oligo. (**B**) Reannealing temperatures normalized to TEL to easily visualize the change in temperature for each sequence.


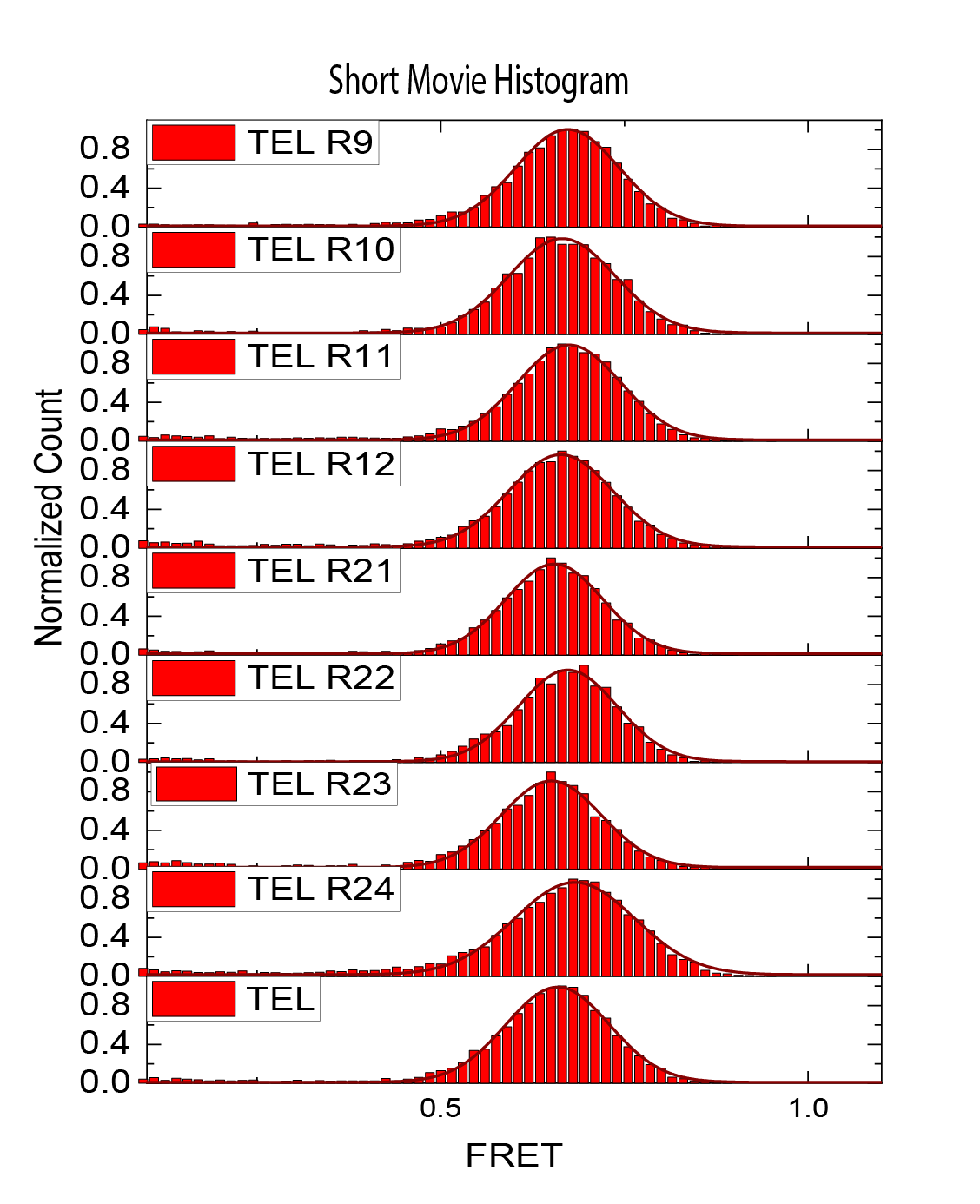


Supplemental Figure 3: Short movie histogram of all tested telomeric DNA with rNMP insertion. TEL represents canonical telomeric DNA without rNMP insertion


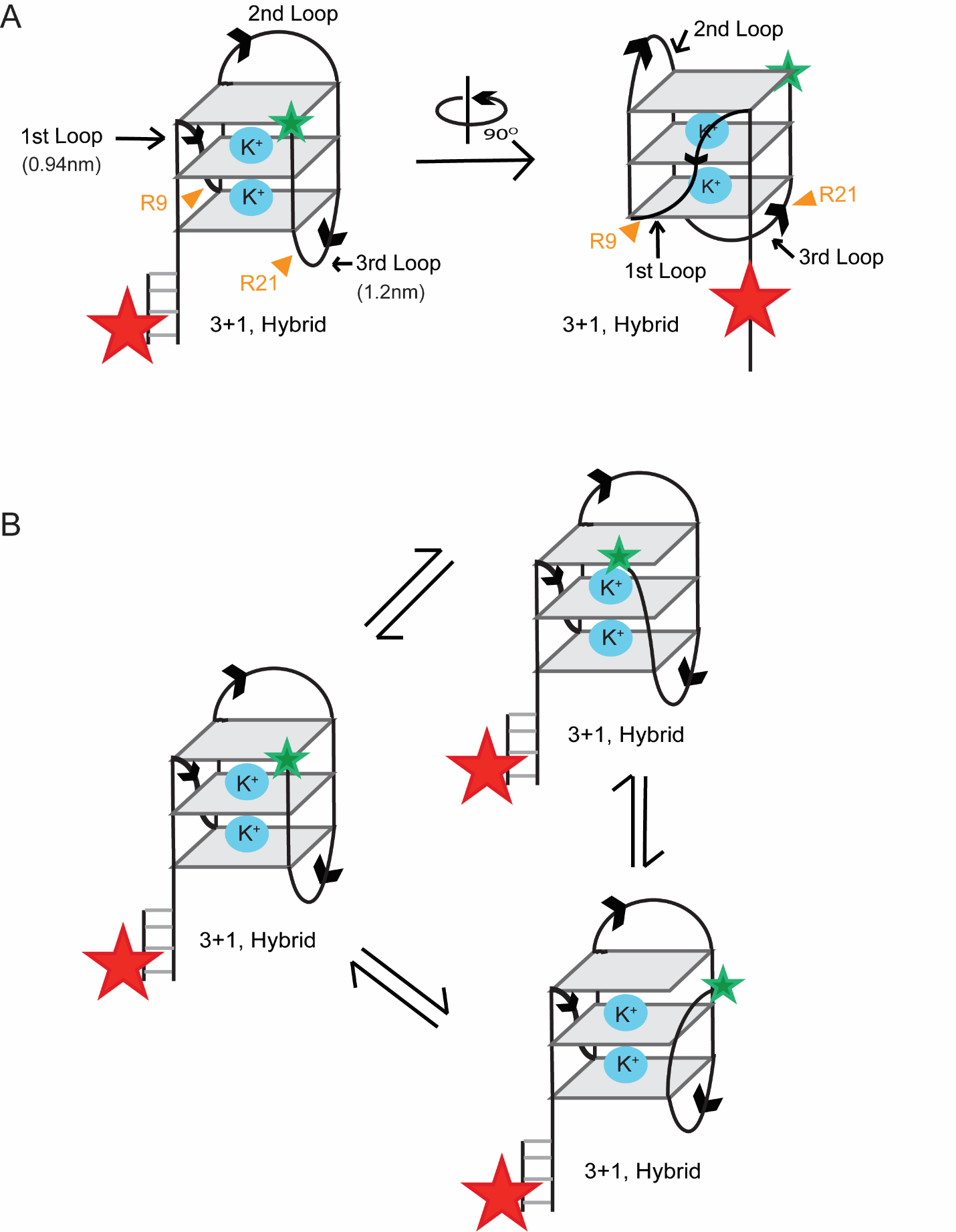


Supplemental Figure S4: The orientation of loops in 3+1 hybrid G4. (A) Different orientation of dye labeled 3+1 Hybrid G4 structure. The number below 1st and 3rd loops are the C5 to C5 distance of each loop measured with PDB 2HY9. (B) Mechanism of flapping. The stand connecting last guanine repeats undergo flapping due to extra hydroxyl group resulting raise of dynamic traces


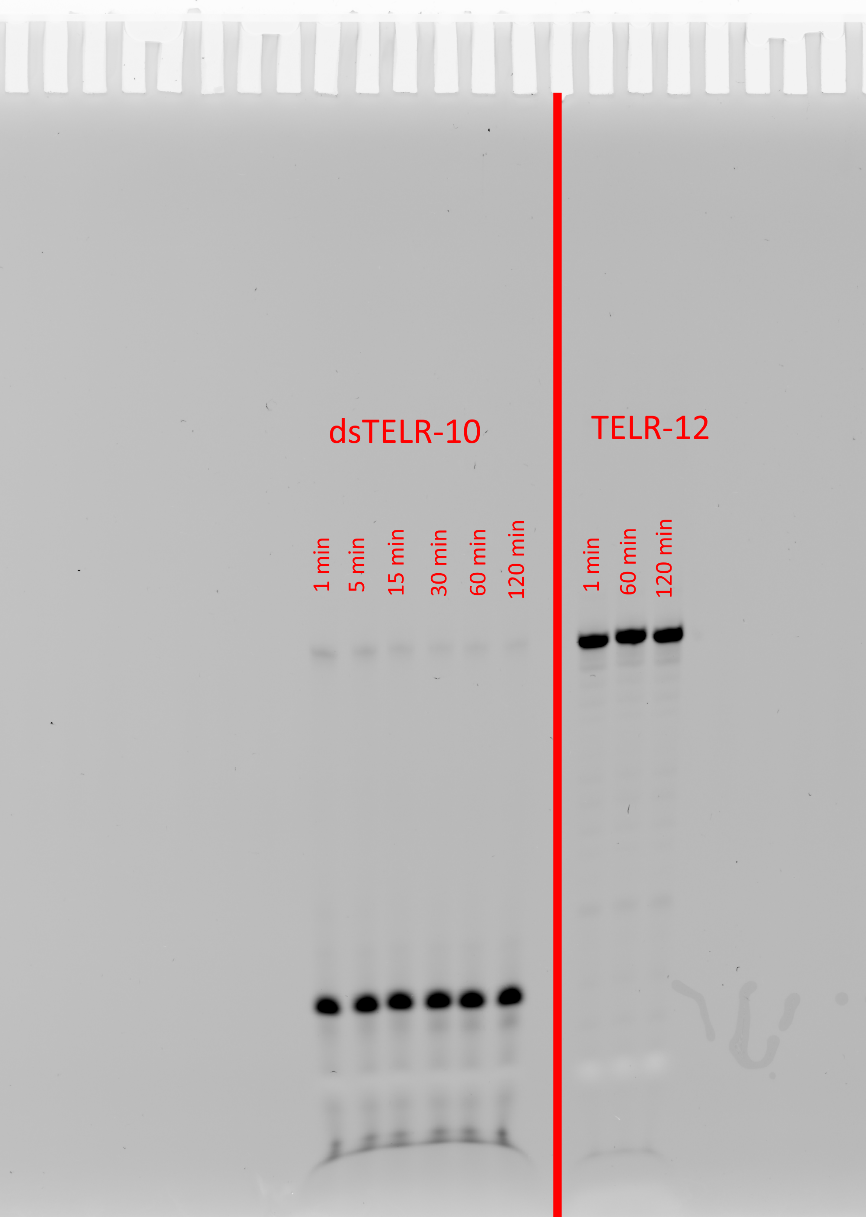


Supplemental Figure 5: Gel with the reactions for dsTELR-10 and TELR-12 in the presence of potassium


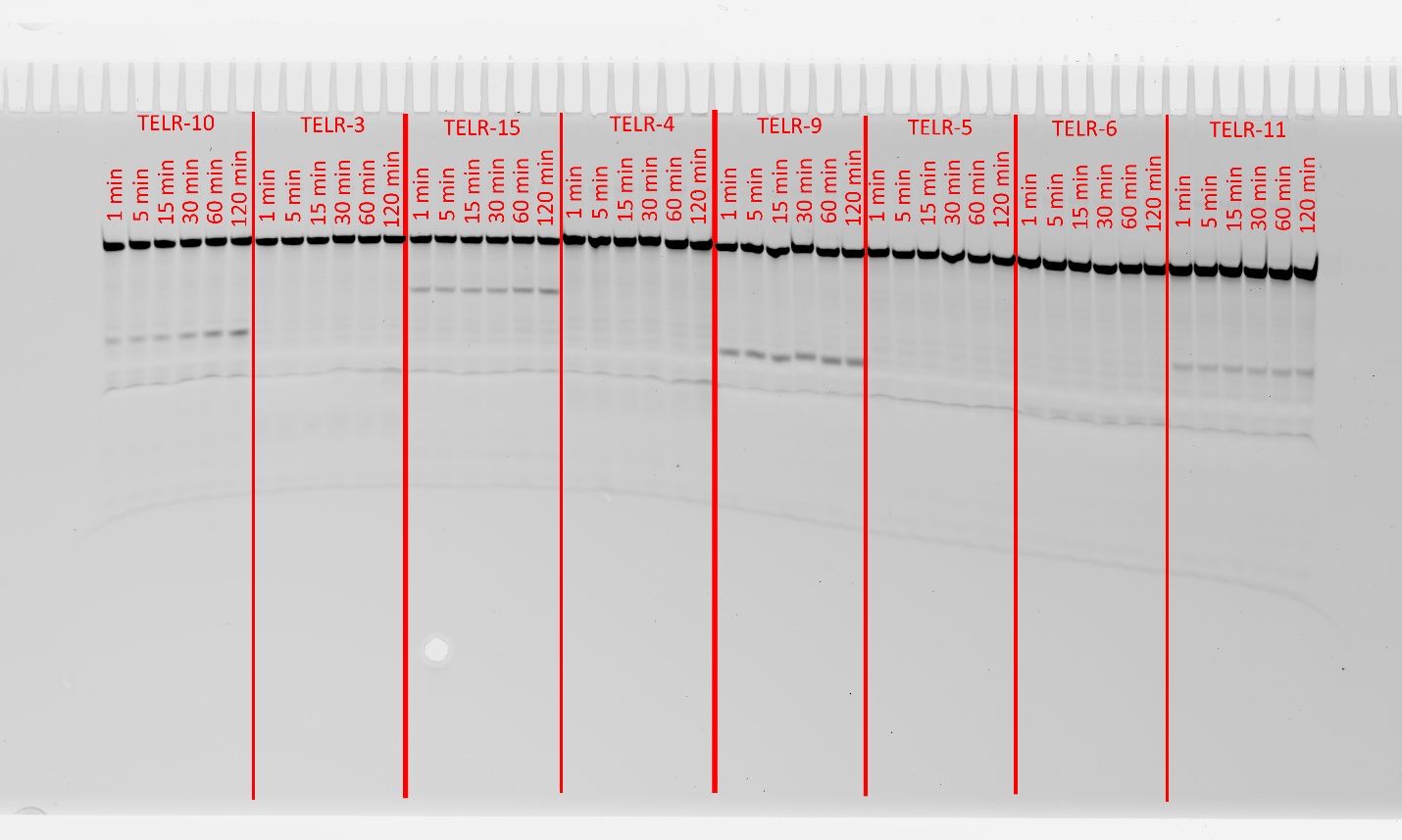


Supplemental Figure 6: Gel with the reactions for TELR-10, TELR-3, TELR-15, TELR-4, TELR-9, TELR-5, TELR-6, and TELR-11 in the presence of potassium
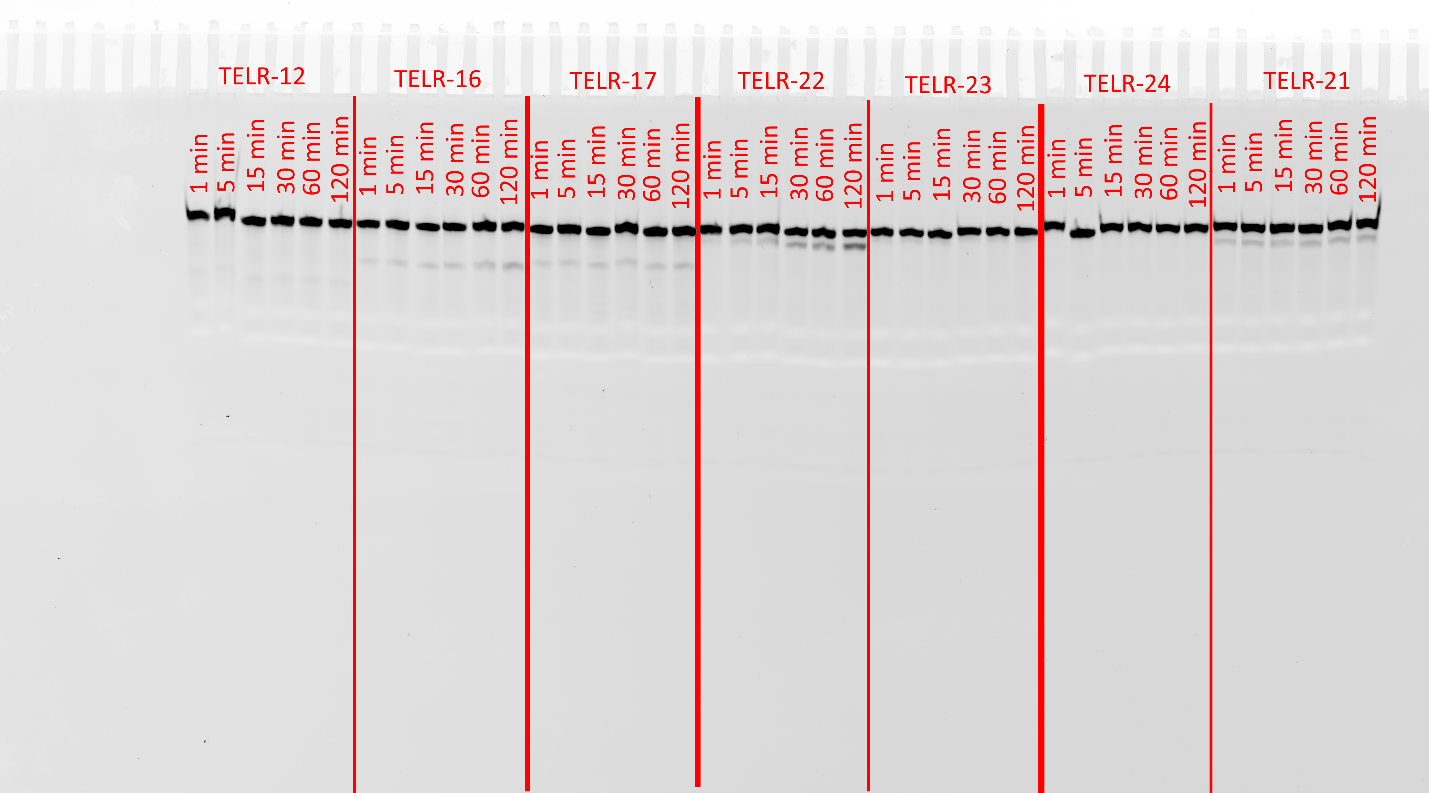


Supplemental Figure 7: Gel with the reactions for TELR-12, TELR-16, TELR-17, TELR-22, TELR-23, TELR-24, and TELR-21 in the presence of potassium
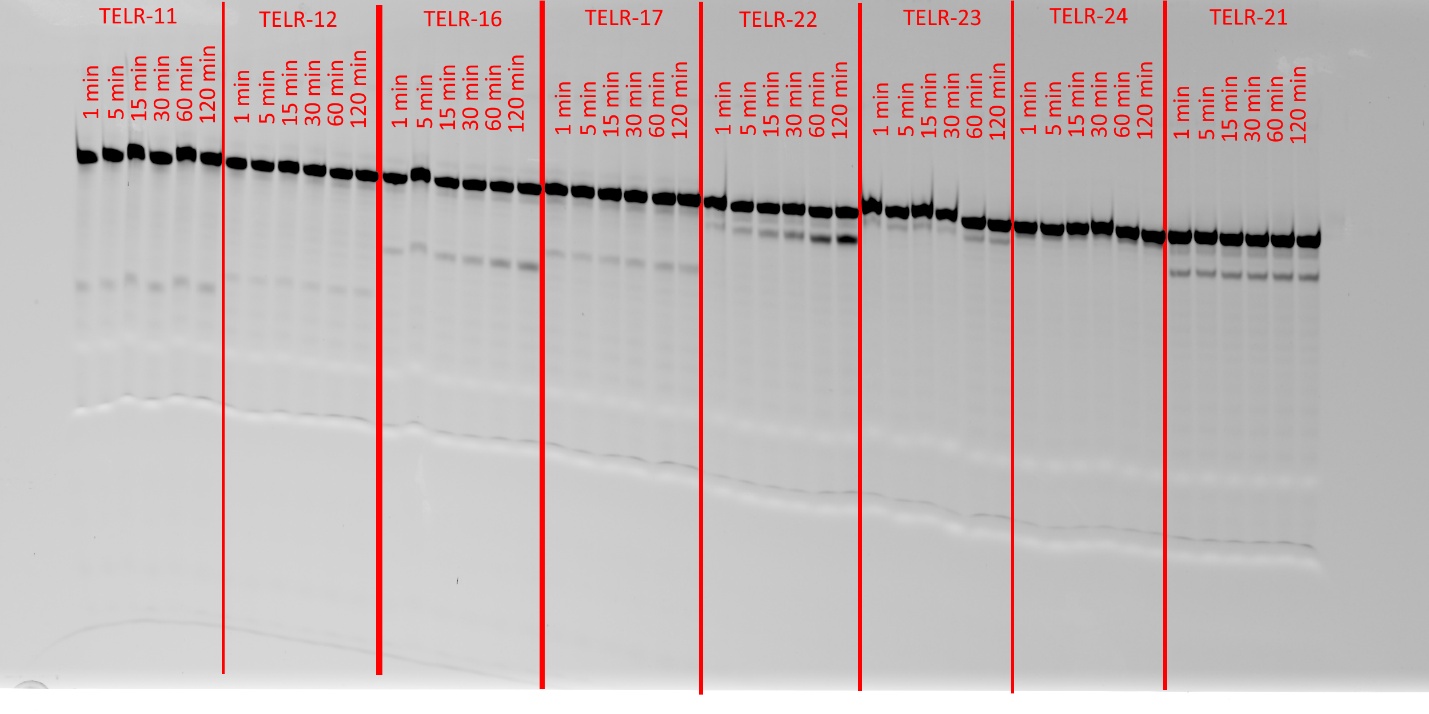


Supplemental Figure 8: Gel with the reactions for TELR-11, TELR-12, TELR-16, TELR-17, TELR-22, TELR-23, TELR-24, and TELR-21 in the presence of potassium
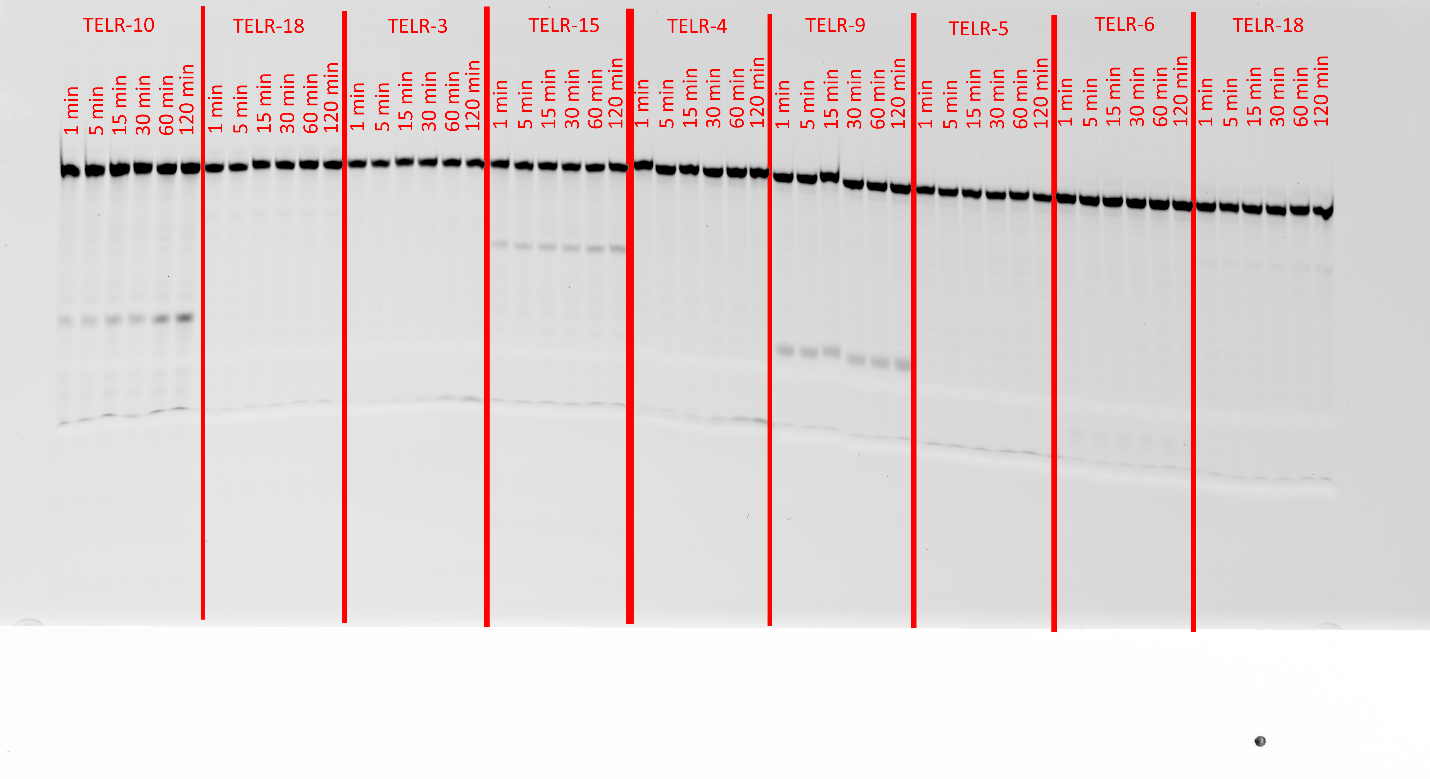


Supplemental Figure 9: Gel with the reactions for TELR-10, TELR-18, TELR-3, TELR-15, TELR-4, TELR-9, TELR-5, TELR-6, and TELR-18 in the presence of potassium


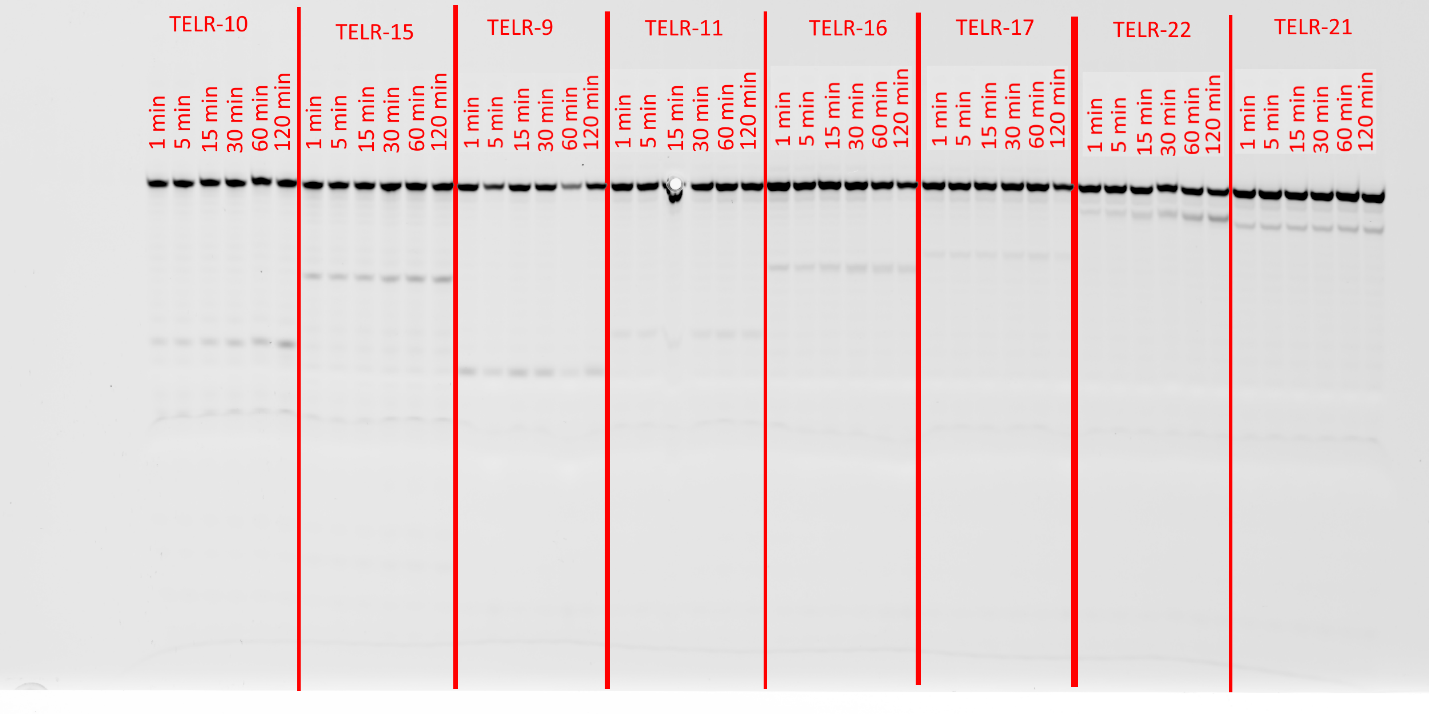


Supplemental Figure 10: Gel with the reactions for TELR-10, TELR-15, TELR-9, TELR-11, TELR-16, TELR-17, TELR-22, and TELR-21 in the presence of potassium


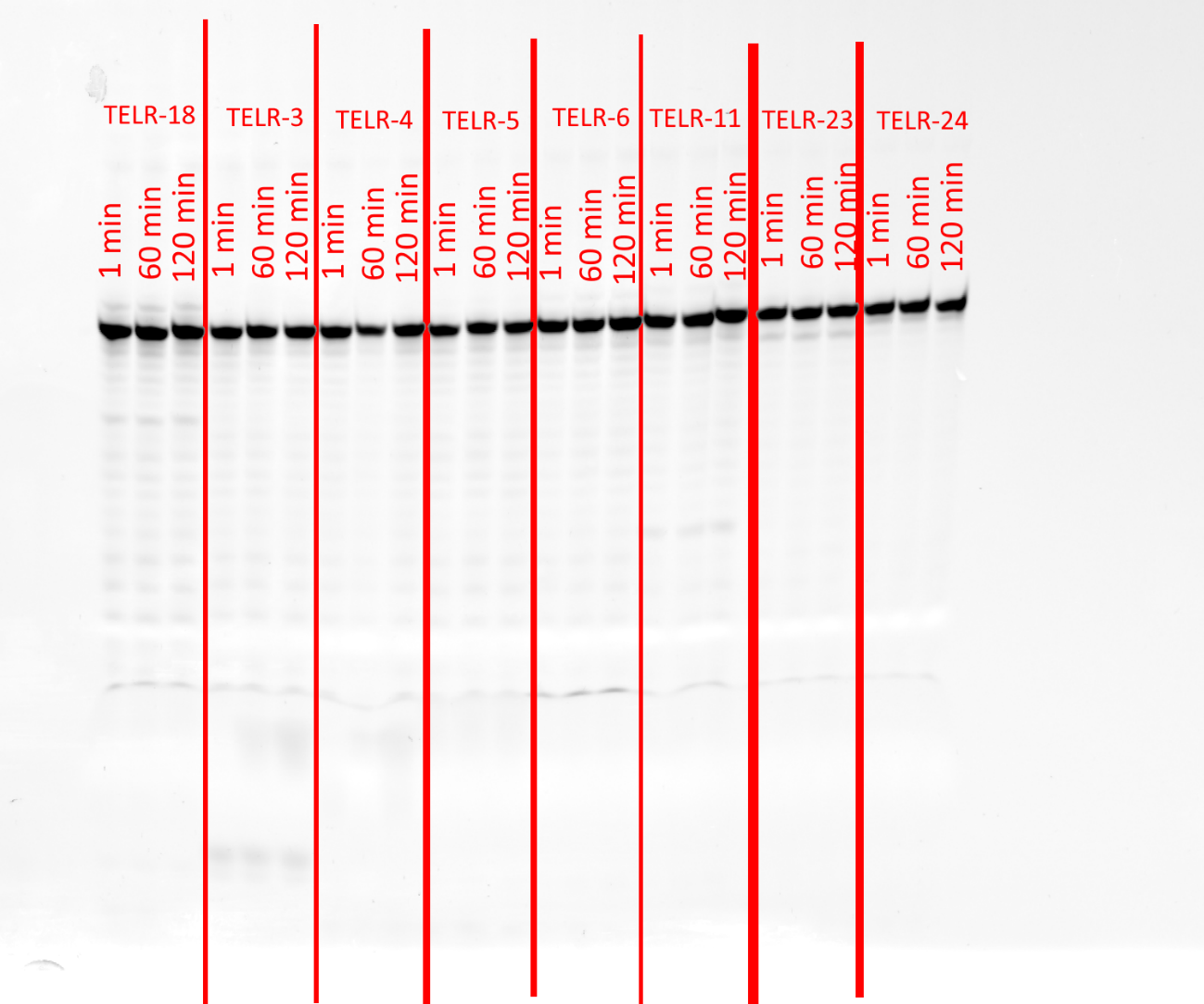


Supplemental Figure 11: Gel with the reactions for TELR-18, TELR-3, TELR-4, TELR-5, TELR-6, TELR-11, TELR-23, and TELR-24 in the presence of potassium


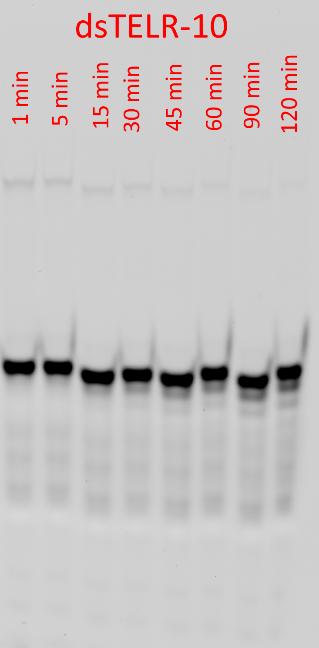


Supplemental Figure 12: Gel with the reactions for dsTELR-10 in the presence of sodium.


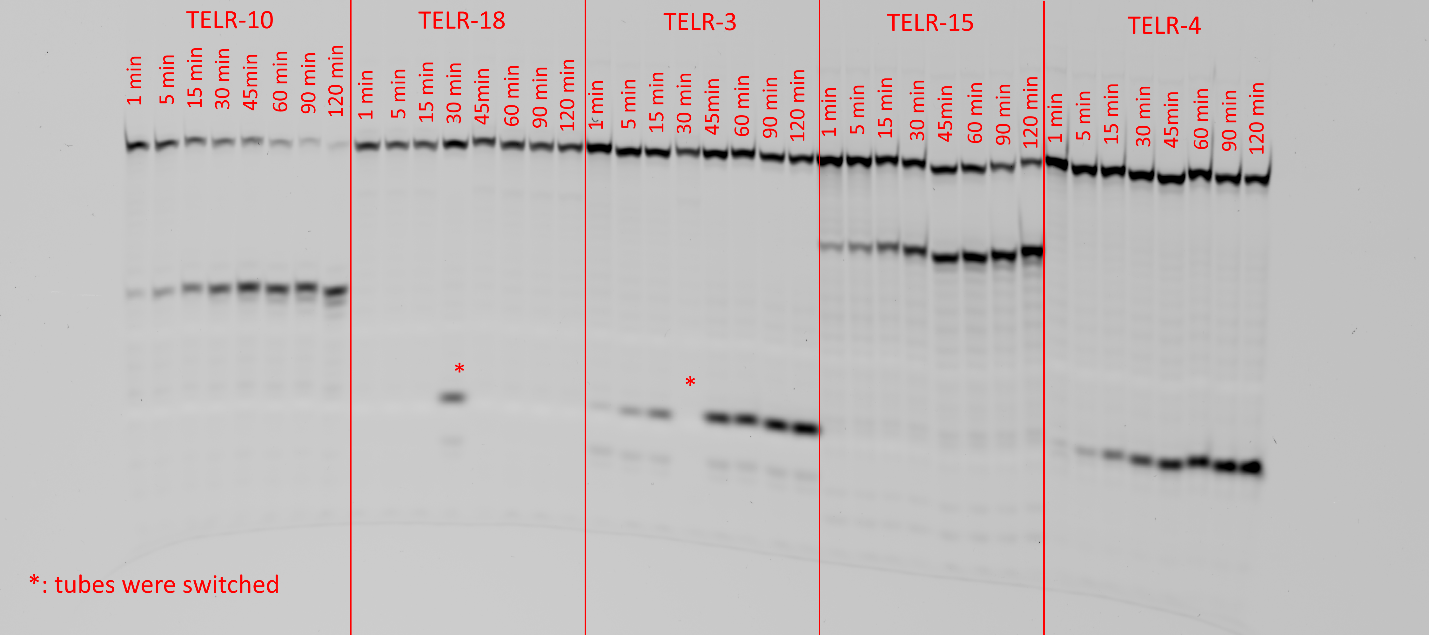


Supplemental Figure 13: Gel with the reactions for TELR-10, TELR-18, TELR-3, TELR-15, and TELR-4 in the presence of sodium.


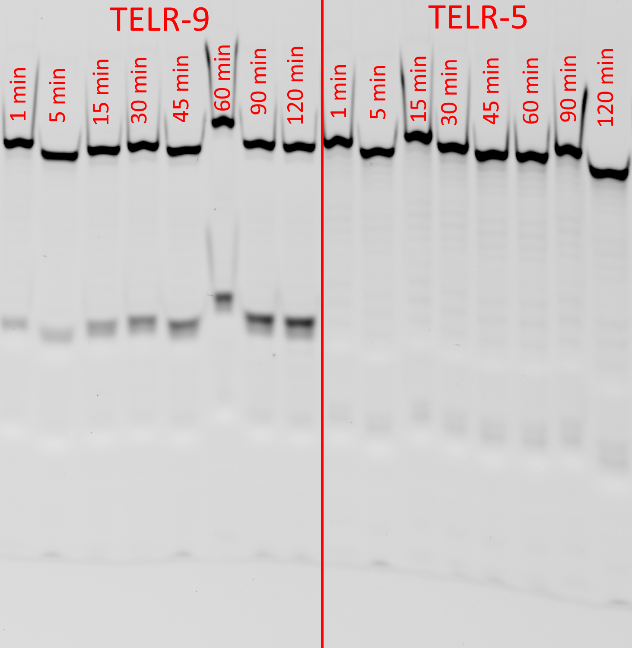


Supplemental Figure 14: Gel with the reactions for TELR-9, and TELR-5 in the presence of sodium.


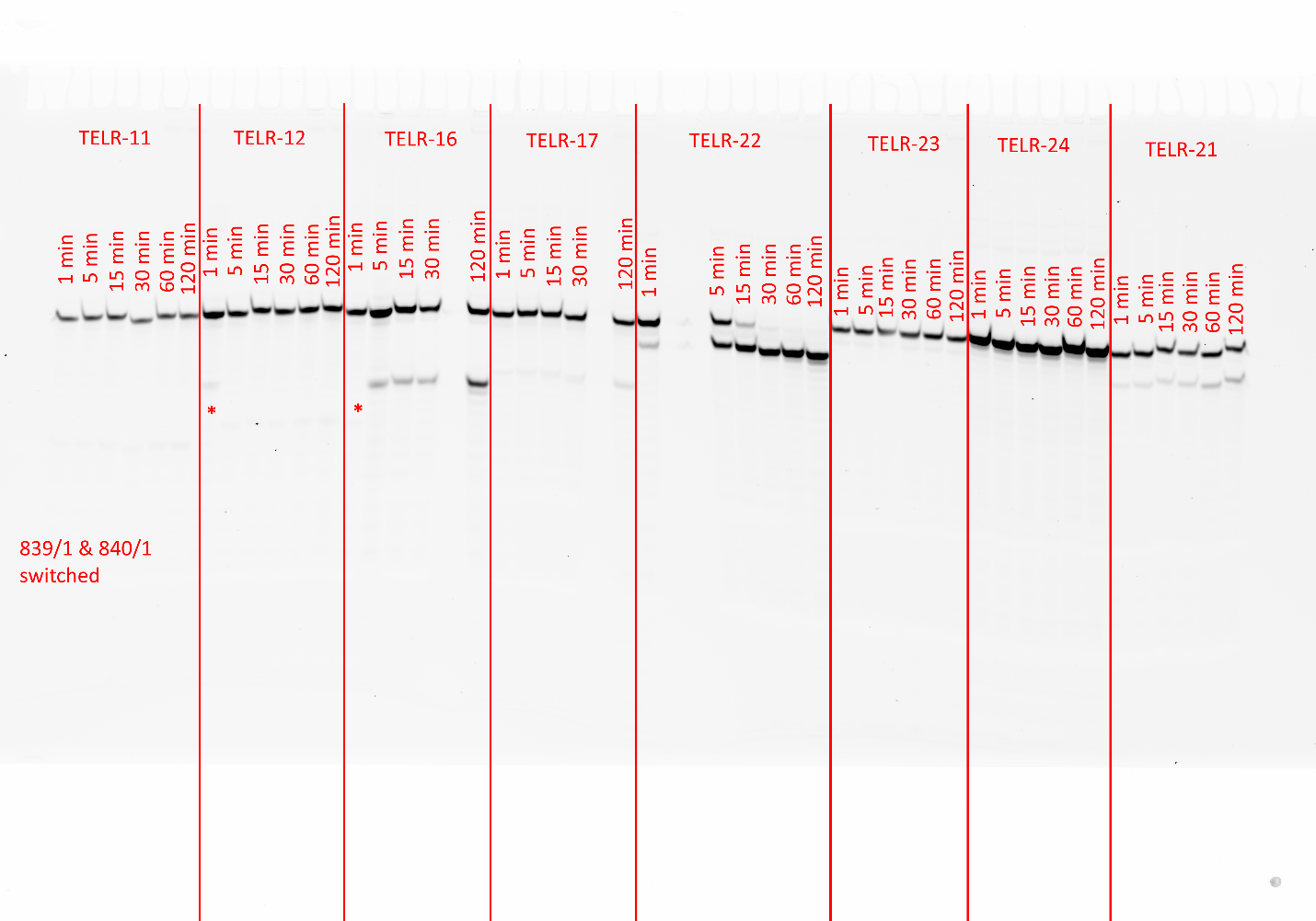


Supplemental Figure 15: Gel with the reactions for TELR-11, TELR-12, TELR-16, TELR-17, TELR-22, TELR-23, TELR-24, and TELR-21 in the presence of sodium.


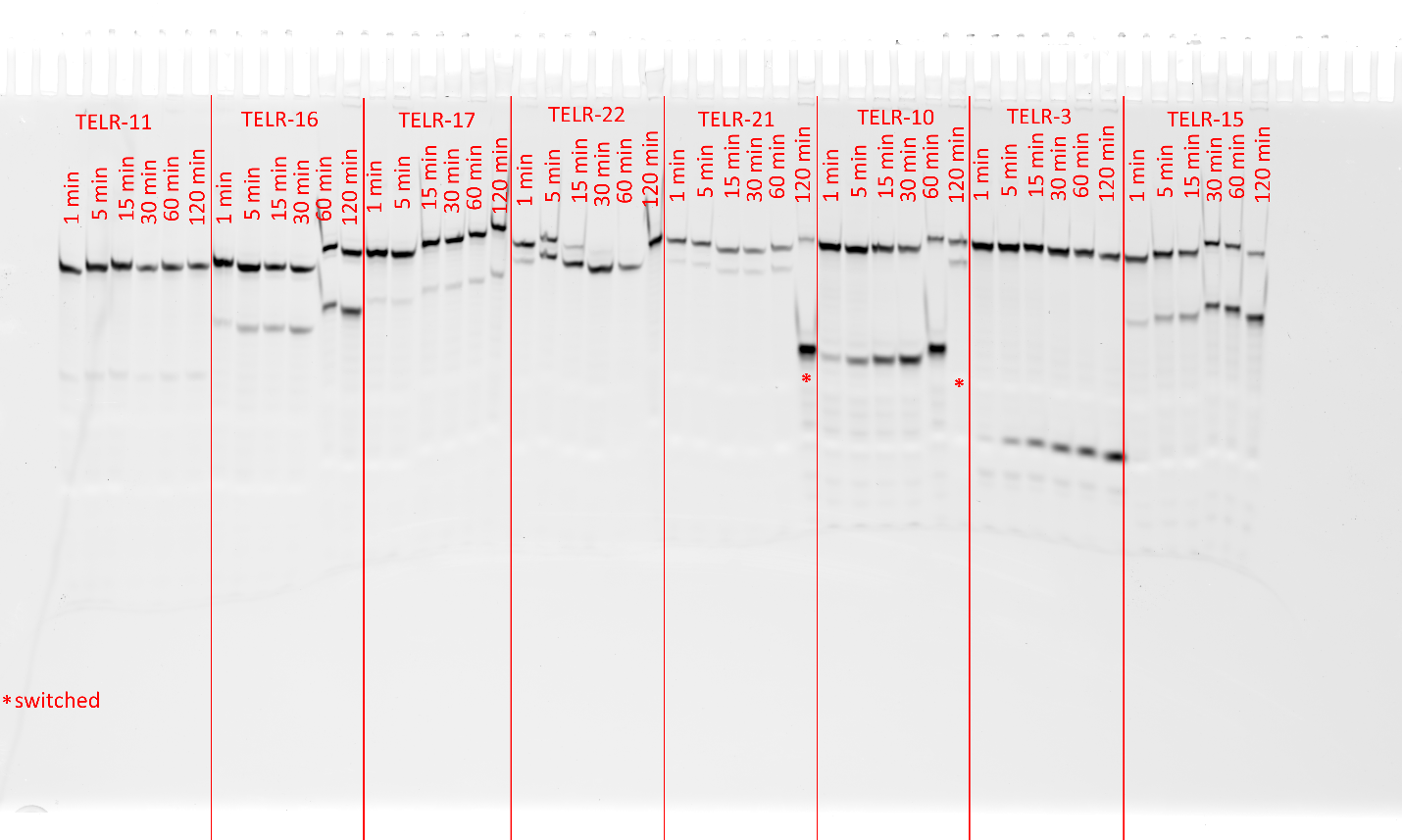


Supplemental Figure 16: Gel with the reactions for TELR-11, TELR-16, TELR-17, TELR-22, TELR-21, TELR-10, TELR-3, and TELR-15 in the presence of sodium.


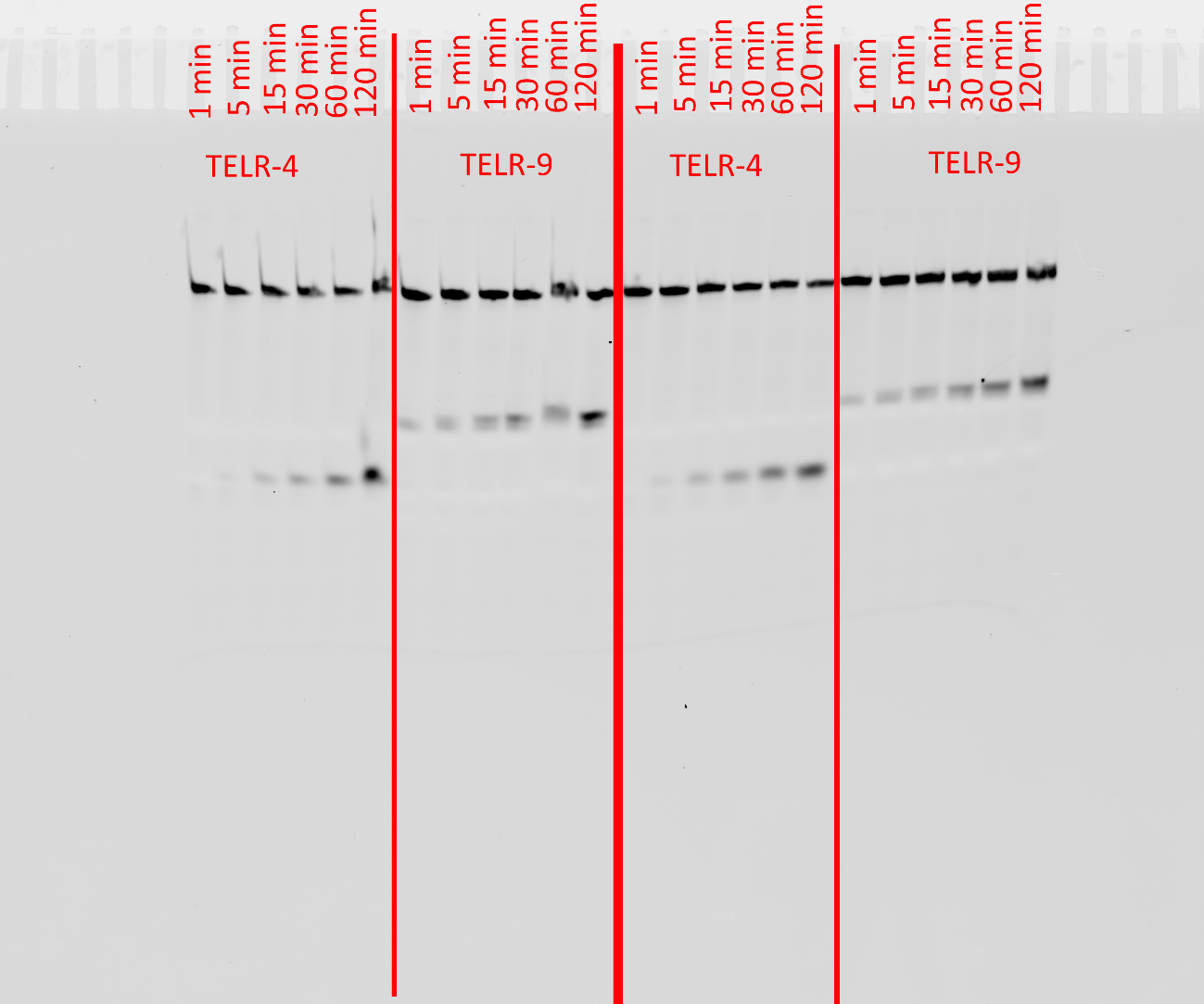


Supplemental Figure 17: Gel with the reactions for two duplicates of TELR-4 and TELR-9 in the presence of sodium.


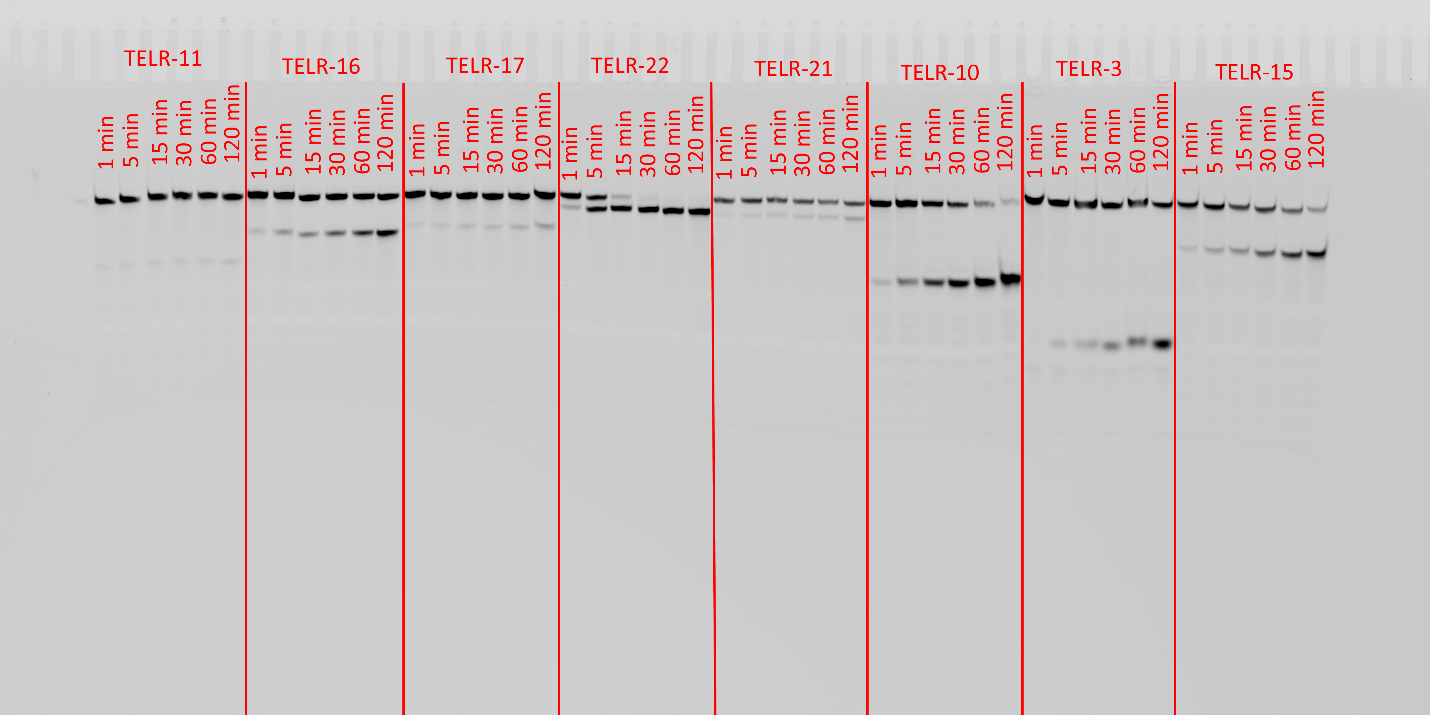


Supplemental Figure 18: Gel with the reactions for TELR-11, TELR-16, TELR-17, TELR-22, TELR-21, TELR-10, TELR-3, and TELR-15 in the presence of sodium.


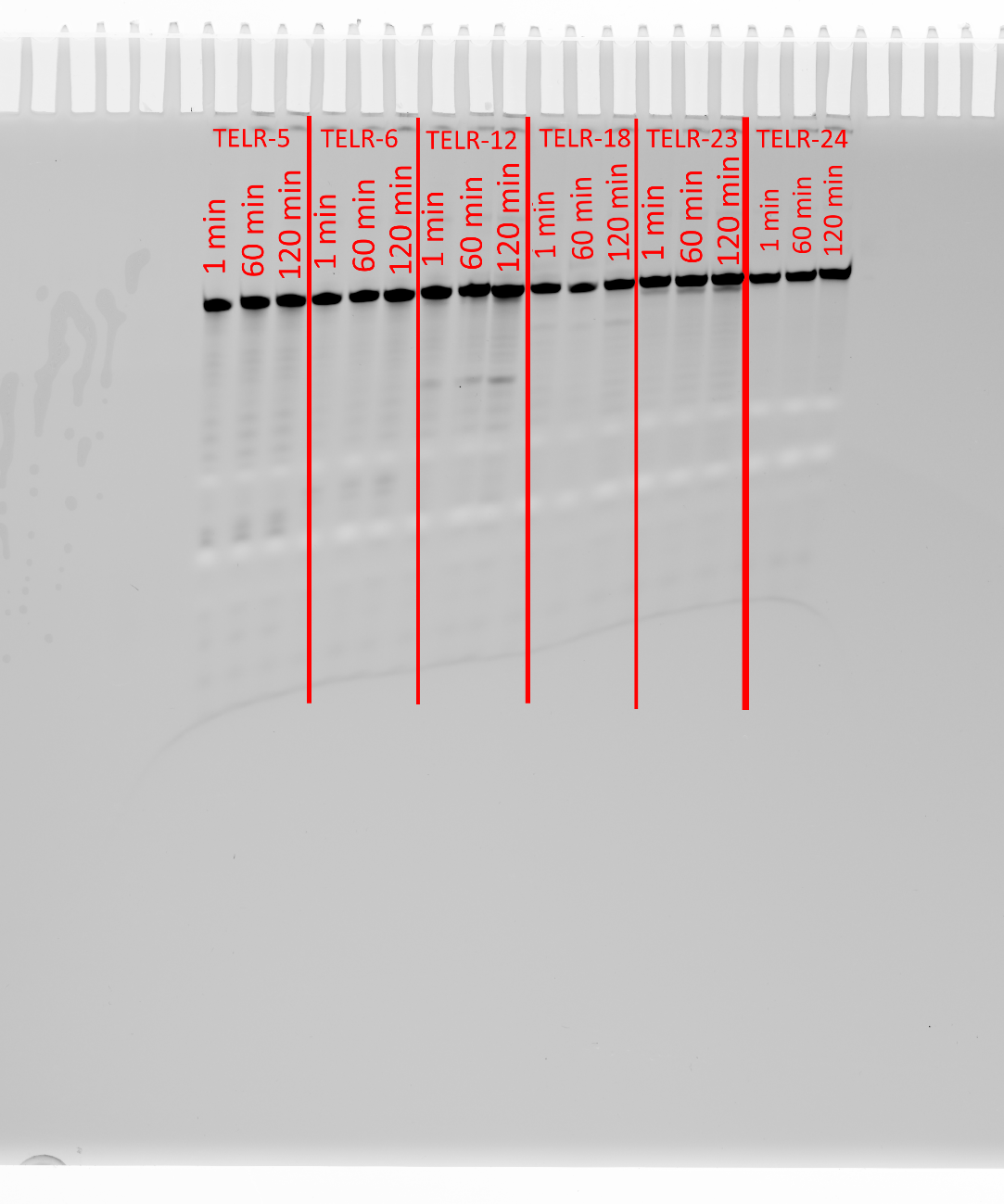


Supplemental Figure 19: Gel with the reactions for TELR-5, TELR-6, TELR-12, TELR-18, TELR-23, and TELR-24 in the presence of sodium.


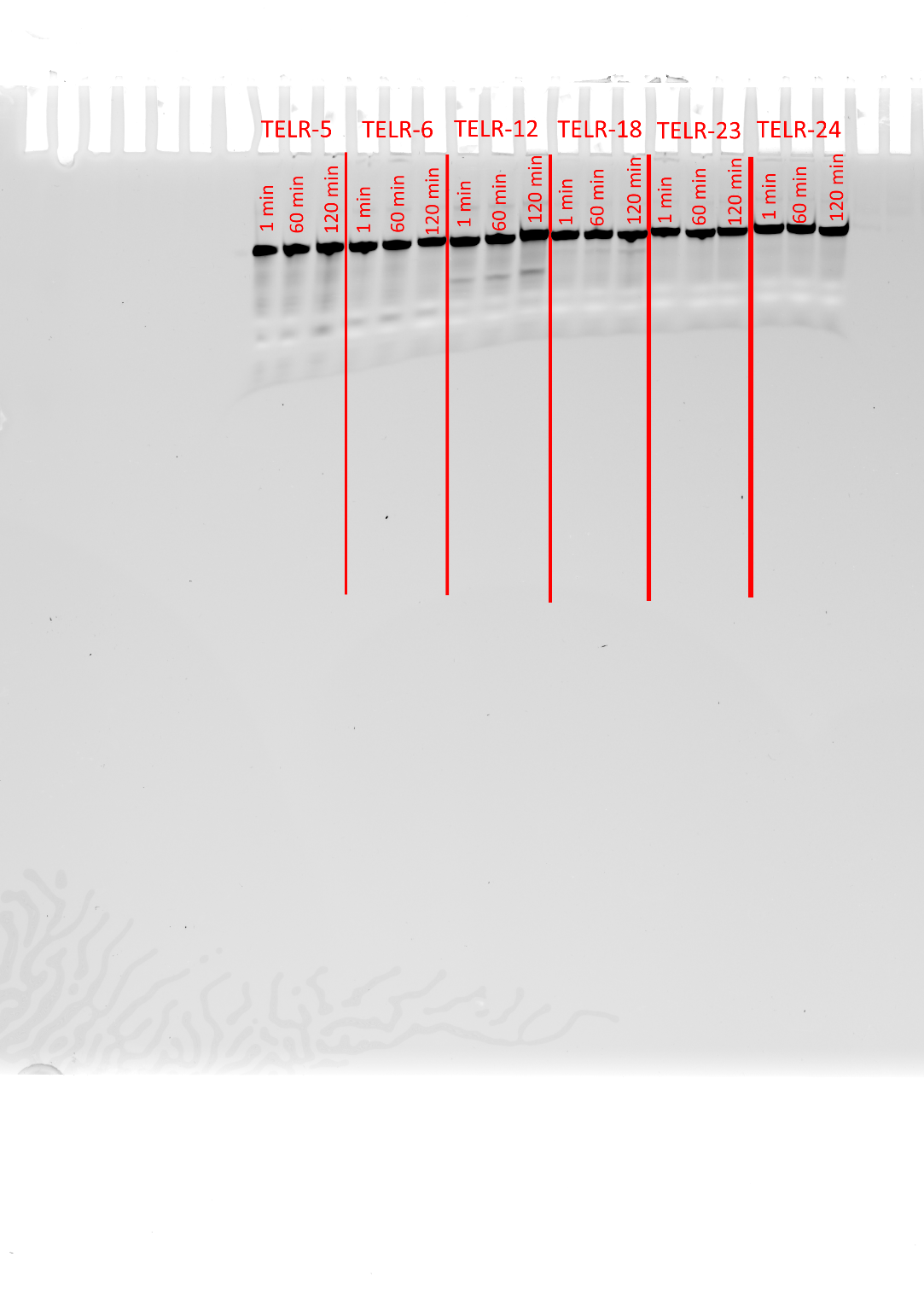


Supplemental Figure 20: Gel with the reactions for TELR-5, TELR-6, TELR-12, TELR-18, TELR-23, and TELR-24 in the presence of sodium.


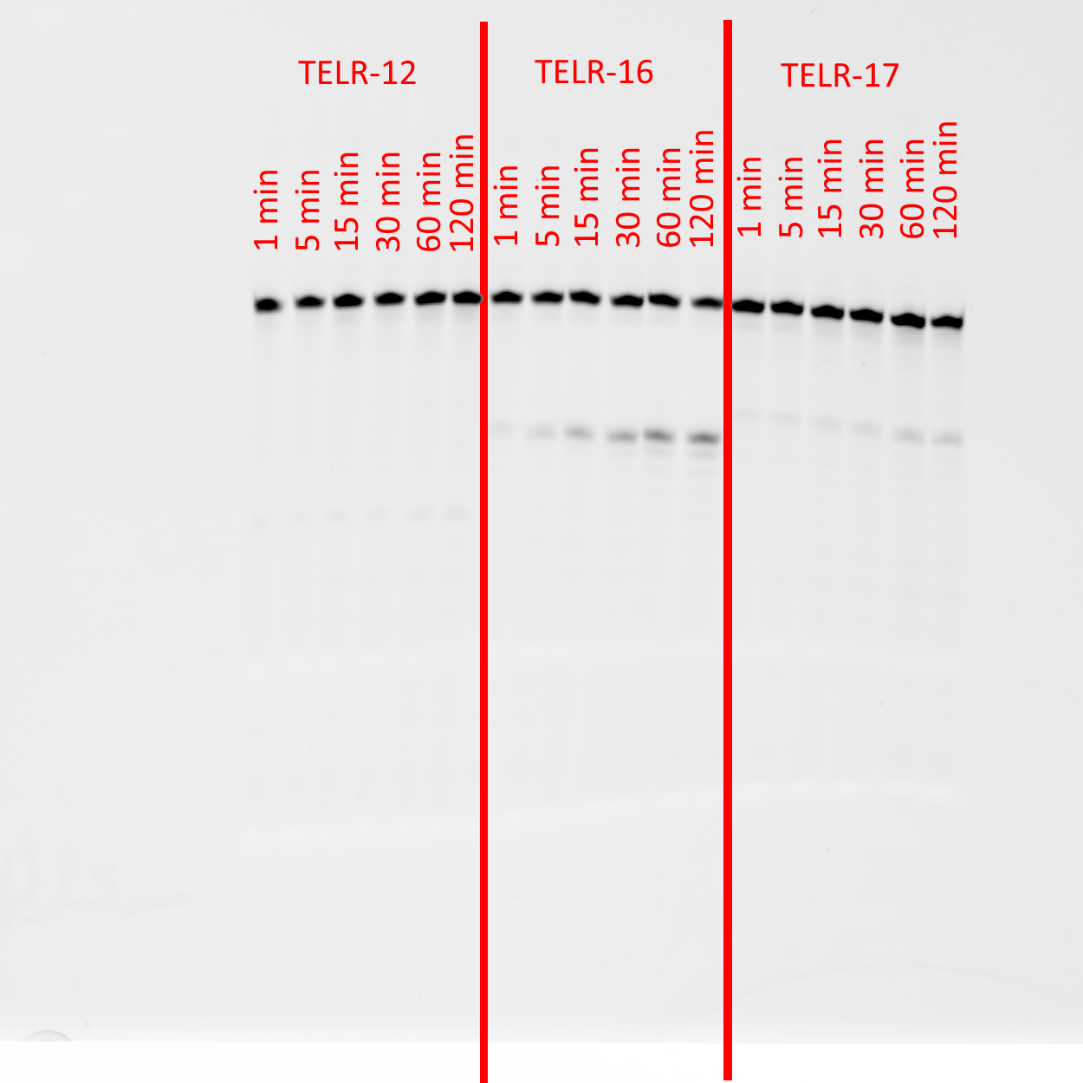


Supplemental Figure 21: Gel with the reactions for TELR-12, TELR-16, and TELR-17 in the presence of sodium.


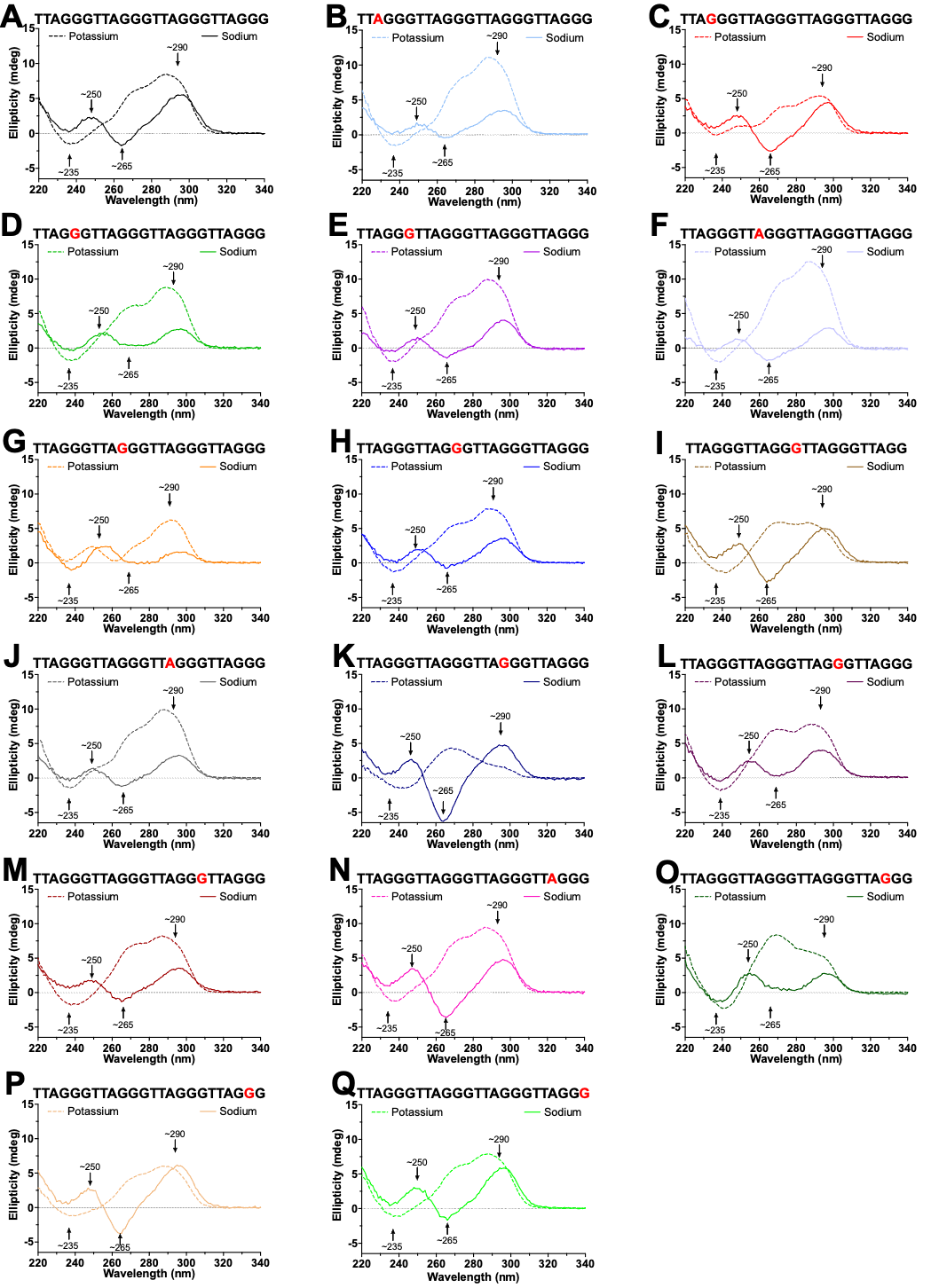


Supplemental Figure 22. CD Characterization of TEL sequences in sodium. Dashed line in each panel represents the original CD spectrum in potassium (Figure 1) solid line in each panel represents the spectrum in sodium. (**A-Q)** 10 CD scans performed at 23°C from 220 nm to 340 nm in 100 mM NaCl and 50 mM Tris with oligos at a concentration of 100 μM. All samples displayed maxima at ~250 nm and ~290 with minima at ~235 nm and ~265 nm which is consistent with antiparallel basket G4 conformation. (**C, I, K, N, P**) These samples displayed a stronger ellipticity at ~265 nm indicating the presence of antiparallel chair G4 which has a single minimum at ~260 nm.

| Sample | Sequence |
| --- | --- |
| TEL_sm_ | 5’-TGGCGACGGCAGCGAGGCTTAGGGTTAGGGTTAGGGTTAGGG/3Cy3Sp/-3’ |
| TELR9_sm_ | 5’-TGGCGACGGCAGCGAGGCTTAGGGTTrAGGGTTAGGGTTAGGG/3Cy3Sp/-3’ |
| TELR10_sm_ | 5’-TGGCGACGGCAGCGAGGCTTAGGGTTArGGGTTAGGGTTAGGG/3Cy3Sp/-3’ |
| TELR11_sm_ | 5’-TGGCGACGGCAGCGAGGCTTAGGGTTAGrGGTTAGGGTTAGGG/3Cy3Sp/-3’ |
| TELR12_sm_ | 5’-TGGCGACGGCAGCGAGGCTTAGGGTTAGGrGTTAGGGTTAGGG/3Cy3Sp/-3’ |
| TELR21_sm_ | 5’-TGGCGACGGCAGCGAGGCTTAGGGTTAGGGTTAGGGTTrAGGG/3Cy3Sp/-3’ |
| TELR22_sm_ | 5’-TGGCGACGGCAGCGAGGCTTAGGGTTAGGGTTAGGGTTArGGG/3Cy3Sp/-3’ |
| TELR23_sm_ | 5’-TGGCGACGGCAGCGAGGCTTAGGGTTAGGGTTAGGGTTAGrGG/3Cy3Sp/-3’ |
| TELR24_sm_ | 5’-TGGCGACGGCAGCGAGGCTTAGGGTTAGGGTTAGGGTTAGGrG/3Cy3Sp/-3’ |
| TEL-TTAG | 5’-TGGCGACGGCAGCGAGGCTTAGGGTTAGGGTTAGGGTTAGGG/iCy3/TTAG-3’ |
| top4.5G18mer Cy5bio | 5’-GCCT/iCy5/CGCTGCCGTCGCCA/3Bio/-3’ |

Table S1: Oligos used in the smFRET assay
